# Voter information campaigns and political accountability: Cumulative findings from a preregistered meta-analysis of coordinated trials

**DOI:** 10.1126/sciadv.aaw2612

**Published:** 2019-07-03

**Authors:** Thad Dunning, Guy Grossman, Macartan Humphreys, Susan D. Hyde, Craig McIntosh, Gareth Nellis, Claire L. Adida, Eric Arias, Clara Bicalho, Taylor C. Boas, Mark T. Buntaine, Simon Chauchard, Anirvan Chowdhury, Jessica Gottlieb, F. Daniel Hidalgo, Marcus Holmlund, Ryan Jablonski, Eric Kramon, Horacio Larreguy, Malte Lierl, John Marshall, Gwyneth McClendon, Marcus A. Melo, Daniel L. Nielson, Paula M. Pickering, Melina R. Platas, Pablo Querubín, Pia Raffler, Neelanjan Sircar

**Affiliations:** 1 Department of Political Science, University of California, Berkeley, Berkeley, CA, USA.; 2 Department of Political Science, University of Pennsylvania, Philadelphia, PA, USA.; 3 Department of Political Science, Columbia University, New York, NY, USA.; 4 WZB Berlin Social Science Center, Berlin, Brandenburg, Germany.; 5 School of Global Policy and Strategy, University of California, San Diego, La Jolla, CA, USA.; 6 Department of Political Science, University of California, San Diego, La Jolla, CA, USA.; 7 Government Department, College of William and Mary, Williamsburg, VA, USA.; 8 Department of Political Science, Boston University, Boston, MA, USA.; 9 Bren School of Environmental Science and Management and Department of Political Science, University of California, Santa Barbara, Santa Barbara, CA, USA.; 10 School of International and Public Affairs, Columbia University, New York, NY, USA.; 11 Bush School of Government and Public Service, Texas A&M University, College Station, TX, USA.; 12 Department of Political Science, Massachusetts Institute of Technology, Cambridge, MA, USA.; 13 Development Impact Evaluation, The World Bank, Washington, DC, USA.; 14 Department of Government, London School of Economics and Political Science, London, UK.; 15 Department of Political Science, George Washington University, Washington, DC, USA.; 16 Department of Government, Harvard University, Cambridge, MA, USA.; 17 Institute of African Affairs, German Institute of Global and Area Studies, Hamburg, Germany.; 18 Department of Politics, New York University, New York, NY, USA.; 19 PPGA de Ciência Política, Universidade Federal de Pernambuco, Recife, Pernambuco, Brazil.; 20 Department of Political Science, Brigham Young University, Provo, UT, USA.; 21 Division of Social Science, New York University Abu Dhabi, Abu Dhabi, United Arab Emirates.; 22 Centre for Policy Research, New Delhi, India.; 23 Ashoka University, Sonipat, Haryana, India.

## Abstract

Voters may be unable to hold politicians to account if they lack basic information about their representatives’ performance. Civil society groups and international donors therefore advocate using voter information campaigns to improve democratic accountability. Yet, are these campaigns effective? Limited replication, measurement heterogeneity, and publication biases may undermine the reliability of published research. We implemented a new approach to cumulative learning, coordinating the design of seven randomized controlled trials to be fielded in six countries by independent research teams. Uncommon for multisite trials in the social sciences, we jointly preregistered a meta-analysis of results in advance of seeing the data. We find no evidence overall that typical, nonpartisan voter information campaigns shape voter behavior, although exploratory and subgroup analyses suggest conditions under which informational campaigns could be more effective. Such null estimated effects are too seldom published, yet they can be critical for scientific progress and cumulative, policy-relevant learning.

## Teaser

A preregistered meta-analysis of six field experiments finds no evidence overall that information campaigns shape voter behavior.

## INTRODUCTION

Voters often have limited information about the performance of their political representatives. These information gaps may undermine democratic accountability: According to many accounts, officials whose actions are shielded from public scrutiny are less responsive to constituents’ concerns and more likely to engage in corruption (*1*). Providing reliable performance information prior to elections may allow voters to select politicians who are more likely to serve them well (*2*, *3*).

Civil society groups and international donors therefore seek to remedy information deficits. For example, the National Democratic Institute has invested in over 15,000 civil society groups globally, with the goal of deepening citizens’ democratic participation by increasing political knowledge. Typical interventions package factual, objective information about politicians’ behavior in easily understood formats and disseminate it to voters via information technology and door-to-door canvassing. See, for instance, the Voting Information Project in the United States, funded by Pew Charitable Trusts, International IDEA’s democratic accountability efforts, or the Transparency and Accountability Initiative (*4*, *5*).

Are these voter information campaigns effective? While information may allow voters to sanction politicians’ poor performance, other theories suggest that voters may not update their beliefs readily in response to new information (*6*–*10*). Experimental studies paint a mixed picture (*5*, *11*–*14*). Yet, previous research may suffer from three difficulties that generally hinder the accumulation of evidence in the social sciences: limited replication, heterogeneity of measurement and design, and publication biases (*15*–*22*). These challenges foster reliance on single, high-profile studies in particular contexts and make it infeasible or unwise to pool data in a formal meta-analysis. The dissemination of studies finding positive effects, but more rarely those showing null results, runs the risk of overstating the efficacy of typical interventions. The published literature, thus, does not readily permit generalizable conclusions about the effects of efforts to alleviate information deficits.

### The Metaketa Initiative: Cumulative learning through collaboration

To address these recurrent problems, we collectively implemented a new approach to cumulative learning. Seven independent research teams coordinated on the design of randomized trials in six developing countries, where information deficits are acute. The studies focused on a common research question and closely harmonized theory, measurement, and estimation. Each study included an intervention that was coordinated across the studies, as well as one or more study-specific interventions. Through this inclusion of both “common arm” and study-specific treatments, we sought to address disincentives that researchers might face in coordinating and replicating interventions. Thus, this structure seeks both to bolster cumulative learning, through coordination on the common arm, and to allow for innovation and analysis of comparative effectiveness through the study-specific arms. Given the premium placed on novelty and differentiation in much scientific work, preserving distinctions between studies while also promoting commonalities may be critical for inducing researchers’ interest in this approach.

We jointly preregistered our meta-analysis of effects from the seven studies before fielding interventions, which is unusual for multisite trials in the social sciences. To limit publication biases, we also committed to integrated publication of results regardless of the findings. Our cluster of studies comprised the inaugural project of the Metaketa Initiative, organized by the Evidence in Governance and Politics (EGAP) network, which seeks to incentivize replication, ensure coordination between researchers to enhance aggregation of findings, and encourage design and reporting standards that guard against selective reporting and publication bias (*metaketa* is a Basque word meaning “accumulation”). Our approach is related to the innovative multisite experiments of Banerjee *et al.* (*23*), who did not, however, preregister their meta-analysis of coordinated trials. Table 1 describes central pillars of the initiative.

**Table 1:** Pillars of the Metaketa Initiative.

**Challenges for** **cumulative learning**	**The Metaketa approach**
1. Confounding in observational research	1. Randomized controlled trials (RCTs)
2. Limited external validity of single RCTs	2. Multiple studies in diverse contexts
3. Heterogeneous, scattered findings	3. Meta-analysis with overall finding
4. Diversity of interventions	4. Coordination on common arm intervention
5. Noncomparable measurement that impedes aggregation	5. Harmonized measurement of inputs, outcomes, and controls
6. Researcher incentives for innovation over replication	6. Study-specific interventions preserve innovation and allow analysis of comparative effectiveness
7. Private data	7. Open data and replication code
8. Errors in data or code	8. Third-party data analysis
9. Fishing (data mining, specification searching, and failure to account for multiple hypothesis tests)	9. Preanalysis plans with limited number of specified hypotheses
10. Publication bias	10. Publication of all registered analyses

## DESIGN

In our inaugural Metaketa cluster, field experiments randomized exposure to information about incumbents’ performance in office. Throughout the world and perhaps especially in developing democracies, voters lack access to information about politicians, government performance, and public services. In Dar es Salaam, Tanzania, a recent survey found that 80% of parents with children in primary education were unaware of how their children’s school fared in the latest round of national examinations, 39% did not know whether teachers at the school came to work, and 25% could not say whether the school had toilets (*5*). Graft is ubiquitous in India: More than 65% of citizens report having paid a bribe to access public services over the past year. Yet, dozens of anticorruption activists have been murdered after legally requesting information under the country’s Right to Information Act (*5*). In addition, deadly antigovernment protests in Caracas in 2014 barely appeared on Venezuelans’ television screens. State control of the media ensured that coverage was limited and sanitized (*5*). These knowledge deficits are problematic on both normative and instrumental grounds; many political theorists suggest that an informed electorate is vital to a well-functioning democracy. It stands to reason that without transparency and a steady flow of reliable information, the corridors of power are likely to be filled with “bad types” of politicians who face few incentives to perform their duties, who may steal from the citizens they are supposed to serve, and yet who are nonetheless reelected. For this reason, donors, activists, and nongovernmental organizations have seen greater transparency as a remedy for what ails democracy. They have sought to repackage and disseminate information on politician performance obtained from government audits, publicly available administrative data, official records, and freedom of information requests.

We sought to test the impact of disseminating similar information in our Metaketa cluster. In their common intervention arms, intended for inclusion in the pooled analysis, each study provided objective, nonpartisan performance information privately to individual voters within 2 months prior to an election. The information was disseminated by flyers, text messages, or videos; was attributable to incumbent politicians or their parties; and was benchmarked to the performance of other politicians, either regionally or nationally. The substance of the interventions focused on legislative behavior (Benin), municipal spending irregularities (Brazil), the quality of public services (Burkina Faso), municipal government malfeasance (Mexico), policy positions and professional experience (Uganda 1), and budget irregularities (Uganda 2). A planned seventh study on incumbent criminality (India) did not take place because of implementation challenges. While some theoretical models have highlighted possible differences between the electoral effects of disseminating information about politician actions as opposed to performance outcomes, our indicators generally encompass both elements and highlight how such a sharp distinction may often be impractical. Most research teams partnered with local organizations, including nongovernmental groups interested in remedying information deficits, to design and implement the studies. Researchers also committed to common ethical principles—such as seeking consent from both subjects and electoral authorities and providing only truthful nonpartisan information—that are critical with field experiments on elections. Further details appear in section S1 and will be the subject of a forthcoming book (*5*).

These standard informational interventions mirror those for which donor and transparency organizations routinely advocate. Several of our studies also replicate or extend experimental treatments from the previous research literature. For example, compare work by Bidwell, Casey, and Glennerster to our Uganda 1 study; work by Chong *et al.* (*12*) to our Mexico study; or work by Ferraz and Finan (*11*) to our Brazil study. Further citations and discussion are in (*5*). Consolidating evidence on the effectiveness of this type of voter information campaign is, therefore, critical.

We collectively preregistered hypotheses, measures, estimation strategies, and tests in our meta-preanalysis plan (MPAP; see section S1.1). Our primary outcome measure is a binary variable that takes a value of 1 if a citizen reported voting for the incumbent candidate or party (depending on the study) and 0 if he or she did not; a secondary dichotomous outcome measures individual voter turnout. Each study administered postelection surveys to measure electoral behavior. Three studies used secret-ballot measures of self-reported vote choice, and others verified electoral turnout through factual questions that only voters who had cast a ballot were likely to answer correctly (see section S1.2, table S1). We collected data on intermediate outcomes, such as perceptions of candidate integrity and effort, or the occurrence of politician campaigns in response to negative information; we also measured possible moderators at baseline, such as coethnic and partisan ties between citizens and politicians. Symmetric measurement across studies facilitates pooling of results.

We hypothesized that the effect of information is likely to depend on what voters already know or believe. Most studies used preintervention surveys to measure voters’ prior beliefs and assess whether the provided information would be “good news,” i.e., information that exceeded prior beliefs about politicians’ performance, or “bad news,” i.e., information that fell short of prior beliefs. For example, in Benin, voters were asked in baseline surveys whether they believe their representative to the National Assembly participates in plenary sessions “much more,” “a little more,” “a little less,” or “much less” than other deputies. In Brazil, preintervention surveys asked voters whether an independent auditor had rejected the mayor’s municipal accounts because of financial irregularities. In these and other studies, the experimental interventions then provided information on actual performance on the same scale used to measure the prior beliefs. Thus, in Benin, politicians in the 75th to 100th percentile of participation were said to participate much more, those in the 50th to 75th percentile participate a little more, and so forth. Examples are included in section S1.3. To construct the information on politician performance, studies relied on official reports of the National Assembly (Benin), third-party audits (Brazil, Mexico, and Uganda 2), expert assessments of candidates’ attributes and policy positions (Uganda 1), and an independent municipal government performance survey carried out by the researchers (Burkina Faso).

**Fig. 1:**
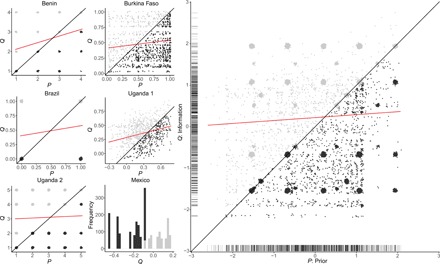
Prior beliefs and politician performance. The figure plots performance information (*Q*) against prior beliefs (*P*) in each of the studies (**left**) and across all studies (**right**). Voters are in the good news group (gray) if information exceeds priors (*Q* > *P*) or if it confirms positive priors (*P* = *Q*, and *Q* is greater than median); otherwise, they are in the bad news group (black). On the right side, *P* and *Q* are standardized with a mean of 0 and an SD of 1 in each study. The density of the dotted areas is proportionate to the number of voters at each value of *P* and *Q*; for the pooled analysis, the rugs along the horizontal and vertical axes indicate the distribution of values. The Mexico study lacked a preintervention survey; thus, we determine the good news and bad news groups according to whether *Q* is greater than the median. The red lines indicate the linear fit between priors and information. For the pooled analysis, the slope of the fit is 0.071; the correlation is 0.053.

Using raw data from the baseline surveys and interventions, Fig. 1 plots performance information (*Q*) against prior beliefs (*P*) in each of the six completed studies (left side of the figure) as well as combined across studies (right side). The density of the dotted areas is proportionate to the number of voters at each value of *Q* and *P*. As we prespecified in our MPAP in section S1.1, news is considered good if information exceeds priors (*Q* > *P*) or if it confirms positive priors (*Q* = *P*, and *Q* is greater than the median value); otherwise, it is bad. Thus, the good news group lies above the 45° line in the plots in Fig. 1, while the bad news group lies below. In the case of the one study, Mexico, that lacked a baseline survey, the figure shows the distribution of politician information; we consider voters to be in the good news group if *Q* is greater than the median value. Figure 1 underscores several related points. First, prior perceptions of politicians are substantially varied, both within and across the good and bad news groups. Second, voters in the bad news group do tend to have more positive prior perceptions of their representatives (although priors do not alone determine bad or good news, since that depends as well on the performance information), and the figure indicates a positive relationship between voters’ prior assessments of politicians’ performance and objective measures of their performance. Last, however, this relationship is quite weak (the correlation between *Q* and *P* across all studies is *r* = 0.053). Thus, there is substantial scope for voters to update their beliefs in light of factual information about politicians’ behavior.

Our core preregistered hypotheses were simple: Positive information (good news) increases voter support for politicians, while negative information (bad news) decreases support. We also expected the effect of information to be increasing in the gap between information *Q* and priors *P*. We theorized that these impacts would operate by changing beliefs about candidates’ integrity and work ethic and would be strongest for nonpartisan and non-coethnic voters. If information campaigns are to reduce information deficits and boost accountability, then these basic effects on voters’ beliefs and behaviors are crucial.

To test these hypotheses in our meta-analysis, we estimate the average causal effect of information for two groups of voters: those for whom the information would be (a) good news and (b) bad news, depending on their priors. Each study randomly assigned the provision of information within each group, often further blocking randomization on background characteristics. To estimate effects, we rely on block-average differences of means, estimated using ordinary least squares (OLS) with fixed effects for randomization blocks. Confidence intervals (CIs) are calculated from SEs clustered at the level of randomization, while we use randomization inference to calculate *p*-values. In table S5, we also present regressions that adjust for pretreatment covariates and estimate the interactive effect of treatment, conditional on a measurement of the gap between the information and prior beliefs. The pooled sample consists of 24,007 individual observations in 1330 randomization blocks in six studies.

We are able to calculate, ex post, the statistical power of our meta-analysis using a simulation approach, which takes into account the specific randomization schemes in the different studies and observed variance in outcomes across randomization blocks (see section S1.6). To register a statistically significant result on our primary outcomes with 80% probability, the interventions would have had to change the vote choice of about 5 of every 100 voters and the turnout decision of 4 of every 100 voters. Given that the experimental interventions introduced often new information (per Fig. 1) and therefore could have had important effects on electoral behavior, we interpret these calculations as evidence that null results were not forgone conclusions.

## RESULTS

**Fig. 2:**
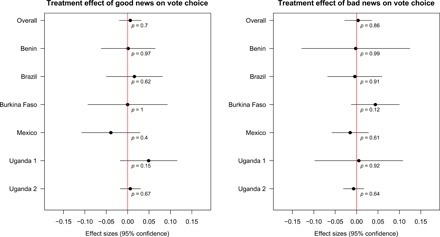
Meta-analysis: Country-specific effects on vote choice. Estimated change in the proportion of voters who support an incumbent after receiving good news (**left**) or bad news (**right**) about the politician, compared to receiving no information. Unadjusted estimates. For estimating the average of the study-specific effects (top row), each study is weighted by the inverse of its size. Horizontal lines show 95% CIs for the estimated change. Entries under each estimate show *p*-values calculated by randomization inference. In all cases, the differences are close to zero and statistically insignificant.

**Fig. 3:**
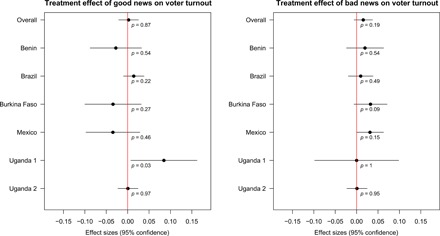
Meta-analysis: Country-specific effects on turnout. See notes to Fig. 2. In all but one test, the differences are close to zero and are statistically insignificant, using *p*-values from randomization inference.

Figures 2 and 3 show the average effects of the informational treatments across all completed studies. The overall effects on our primary outcome variable, vote choice, are statistically indistinguishable from zero, both in the good news group (point estimate, 0.62 percentage points; 95% CI, −1.95 to 3.19) and the bad news group (point estimate, 0.36 percentage points; 95% CI, −2.80 to 3.51). We also find no evidence that the impact of treatment assignment interacts with the gap between prior beliefs (*P*) and the information (*Q*) (table S5). As Fig. 3 shows, we also find null overall results for our secondary outcome, voter turnout. In the pooled meta-analysis in Figs. 2 and 3, we estimate the average study-level effect of the treatments across the set of six completed experiments. We also prespecified a secondary Bayesian analysis, in which we assume that each treatment effect is drawn from a common population of effects. This approach involves stronger assumptions than our primary approach, but it allows assessment of the likelihood of effects for each study in light of learning from the other studies (see MPAP in section S1.1). However, this analysis produces similar substantive conclusions (figs. S7 and S8).

Figures 2 and 3 also display study-specific results for each of the six completed experiments using our preregistered specification for the common arm interventions. Despite substantial contextual differences, we cannot reject the null hypothesis for either good or bad news for any single study in the case of vote choice. Moreover, the estimates are close to zero in nearly every study. In two of the three studies where the effect estimates are furthest from zero in absolute value, the statistically insignificant effect appears in the theoretically unexpected direction (a positive effect of bad news in Burkina Faso and a negative effect of good news in Mexico). In the case of turnout, all but one of the study-specific estimates are statistically insignificant according to *p*-values based on randomization inference. Our power to reject the null hypothesis of no effect of the common intervention is much less for individual studies. Yet, similar findings from multiple studies boost our confidence that the overall effect of the standardized voter information campaigns is weak.

**Fig. 4:**
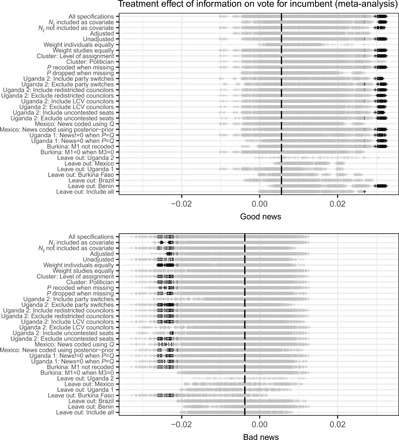
Robustness of findings across specifications: Vote for incumbent. Estimates across all specifications of the overall treatment effect of the common informational intervention on vote for incumbent. The vertical axis lists all considered specification choices. The top row shows the collection of estimates across all specifications. Each subsequent row holds fixed a given specification choice and shows the distribution of treatment effect estimates, varying all other choices. Darkened vertical lines show estimates for which *p* < 0.05. The dashed vertical line indicates the estimated average treatment effect reported in table S5.

**Fig. 5:**
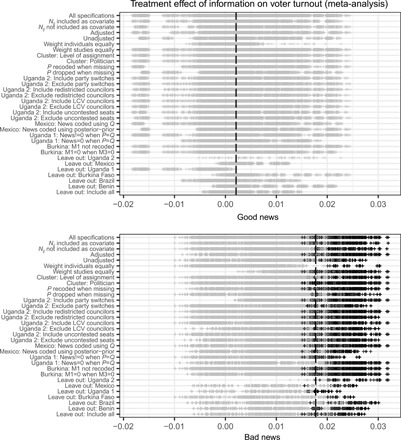
Robustness of findings across specifications: Turnout. See notes to Fig. 4.

How robust are these null findings? Our primary estimations closely follow our registered MPAP and study-specific preanalysis plans. In some cases, the preanalysis plans were not sufficiently specific or could allow for different interpretations, proposed more than one strategy (for instance, inclusion or exclusion of covariates), or research teams found study-specific deviations appropriate. We present a type of specification curve analysis that shows the robustness of results to these unregistered analytic choices. See (*5*) for further discussion of specification curves, as developed by Simonsohn *et al.* (*21*). Thus, we estimate a set of 18,886 model specifications reflecting all possible combinations of these deviations and ex post decisions. Figure 4 shows results from the full set of models for vote choice, while Figure 5 depicts results across specifications for turnout. In each figure, the plot in the top panel shows estimated effects of good news, and the bottom panel shows bad news. In each plot, the horizontal axis depicts the estimated average treatment effect, while the vertical axis lists the set of decisions (see sections S2.2 and S2.3). Decisions come in pairs (e.g., unadjusted versus covariate-adjusted analysis), with the exception of an unregistered “leave-one-out” analysis in which we calculate the overall meta-analysis estimate, excluding one study at a time. Within the row associated with a particular decision, that decision is held fixed, and estimates from all other possible specifications, i.e., specifications based on all combinations of other decisions, are presented. (We exclude sets of specifications that are not plausible, such as choosing to recode a variable in a particular study when that study is excluded from the analysis.) Each vertical dash in the body of the plot denotes a point estimate for a single model. We darken those estimates that are nominally statistically significant at the 0.05 level. The first row of each plot shows the collection of estimates from all specifications and, thus, depicts the overall proportion of estimates that are nominally significant.

We find that the null results are highly robust for the overall effect of the common information arm across the six completed studies. For vote choice, significant effects of good news about politician performance materialize only in 0.3% of specifications; for bad news, the treatment effect estimate is significant in 0.6% of specifications. For turnout, we find significant effects in a larger proportion (10.3%) of bad news specifications, but in no cases do we find evidence of an overall effect of good news. It bears emphasis that these specifications are not a random draw from a set of all possible specifications; rather, they reflect ex post decisions that may move in the direction of estimating significant effects. The analysis also allows us to understand which specific decisions are necessary to obtain significant results in our meta-analysis. For example, specifications in the bad news/vote choice analysis are significant only in the presence of specific choices related to the Uganda 2 study [excluding candidates who switched parties and including Local Council V (LCV) councilors] and when we exclude Burkina Faso from the meta-analysis. The significant specifications in the bad news/turnout case occur over a wider range of choices, with no particular choice decisively needed to obtain significance. See section S2.3 for discussion. In the online materials, we also present a flexible interface (an R-based Shiny app) that allows users to vary specification choices themselves and assess the impact for overall and study-specific results (see http://egap.org/content/metaketa-i-shiny-app).

In addition, we conduct a sensitivity analysis, in which we ask how big (in absolute value) the estimated effect in the uncompleted India study would have needed to be to produce a non-null estimated effect in a seven-study meta-analysis. In the good news case, we would have required an estimated effect of at least 17.2 percentage points, an extremely large effect, much bigger than anything we see in other studies (section S2.4). Unreliability of self-reported voting data also does not likely explain our null effects: Any reporting biases might lead voters in the treatment group to overreport vote choice for incumbents, at least in the good news group, leading us to falsely reject true null hypotheses—rather than fail to reject false nulls.

## DISCUSSION

What explains the weak overall effect on voter behavior of these common, potentially scalable informational interventions? There are many necessary steps in a causal chain linking information to voters’ decision-making process and, ultimately, to greater political accountability (*13*, *24*). In our studies, information existed and was disseminated. The extent to which voters understood the information varied markedly across studies, with nonresults in some studies suggesting real difficulties of disseminating information effectively, a feature that is likely to be a concern with many informational campaigns more broadly (tables S8 and S9). Perhaps most critically, however, the information did not, on average, have any discernible effect on perceptions of politicians’ integrity and effort (table S10). In unregistered analyses, we do not find that this effect interacts with respondents’ perceptions of the credibility of the source of the information (table S11). Our data thus suggest that these standard types of informational campaigns simply did not induce voters to update their beliefs or produce meaningful change in their perceptions.

These results do not imply that informational interventions can never be effective. The structure of our Metaketa, with its distinction between common and study-specific interventions, was designed to allow initial assessment of hypotheses about how modifications of standard information provision campaigns might make them more impactful. In alternative intervention arms, for example, three studies in our cluster provided information to voters in a public setting, rather than privately to individual voters, on the theory that this could generate common knowledge of the intervention and foment coordination between voters. In a preregistered analysis pooling data from these studies, we find a large and significant effect of the informational treatment in the public condition in the good news, but not the bad news, case, albeit driven by a large effect in one study (tables S21 and S22). This could relate to previous nonexperimental as well as experimental findings that the presence of robust media markets can amplify the impact of information (*11*, *25*–*28*). In preregistered study-specific analyses, the Benin study also finds that prompts on the importance of the domain about which information is provided, as well as efforts to augment coordination across villages, can boost the effectiveness of standard informational interventions, and the Uganda 1 study finds that their intervention shapes choices in favor of opposition candidates. When examining precinct-level electoral returns, the Mexico study, which assigned treatment to most households within treated precincts, finds that the provision of performance information increased incumbent vote share, but significantly less so where reported malfeasance was greater. (The authors of the Mexico study regard the precinct-level electoral returns as more reliable, given that the secret ballot technique used to elicit survey-based vote choices was confusing to many voters.) These and other examples are discussed at more length in (*5*). The Uganda 2 study presents evidence of impact of information about district councilors at one level of government but finds no impact of information about district chairpersons (*29*). These study-specific findings are intriguing and should be the subject of further comprehensive research. For example, the effects of public dissemination could be tested rigorously as the common intervention in a new Metaketa cluster.

The structure of our initiative also allows us to test some exploratory findings from one study against data from the collection of studies as a whole. For example, if one variable is an important feature driving effects in one study, then this can be tested systematically using data from other studies. While there is evidence from one of our experiments that coethnicity moderates the effect of information (*30*), overall we do not find that effects vary as a function of coethnic, copartisan, or clientelistic relationships between voters and individual candidates (table S15). We also explored whether local contextual factors, such as the extent of electoral competition, condition the impact of the treatments, but find no evidence that they do (tables S16 and S17). Last, although we detect some signs that politicians may seek to undermine the dissemination of negative information (table S13), a salient feature of the individual studies in Mexico and India, we also find null results in studies where there is no evidence of this backlash. In our case, the consistency of the null estimated effects—despite heterogeneity in the substance of the information and across the contexts in which the experiments were fielded—therefore reinforces confidence in the overall finding.

This mode of out-of-sample testing illustrates a general source of leverage in the Metaketa approach, which may prove helpful in cases where results are more varied across studies than they were in ours. Suppose, for instance, that we had found positive effects in some studies but negative or null effects in others. This would have raised an explanatory challenge, since contexts vary in many ways. It would be difficult or impossible to identify which source of study-level variation accounts for the different estimated effects. However, the Metaketa approach allows for principled ex ante commitment to testing how different dimensions of heterogeneity condition treatment effects, not at the study level but by combining within-study and cross-study information, as in the analysis of our moderating effect of coethnicity. This strategy can substantially ameliorate the “degrees of freedom” problem and could prove important in the analysis of four additional Metaketa clusters that are currently underway. See https://egap.org/metaketa for information on the other Metaketa clusters.

In our Metaketa cluster, the core findings provide an important cautionary note on the limits of using blanket informational interventions to improve democratic accountability. Focusing on our prespecified estimation strategies but also exploring several deviations from these plans, we find no evidence of overall impact of standard, nonpartisan voter information campaigns targeted to individual voters. Exploratory and subgroup analyses, as well as evidence from preregistered study-specific interventions, suggest modifications that could make informational campaigns more effective, and, of course, we cannot rule out the possibility that interventions at a much greater scale could have positive effects. Yet, this evidence suggests that typical transparency promotion efforts, such as those backed by civil society organizations and donors, may not pack a strong enough punch to influence voter behavior. These conclusions should provide useful input for policymakers because the interventions we studied are typical of many ongoing real-world programs. These results also inform basic science: If individual voters are slow to respond to new facts to update their beliefs, then this calls into question the premise of many theoretical models that voters have an appetite for information and use it to exert control over politicians.

Null effects such as those presented here are important for scientific progress and policy-relevant learning, yet they are too rarely published. The consistency of our results underscores the value of replicating similar studies in diverse settings: Despite the heterogeneity of contexts, the estimated effects of the baseline informational interventions are quite uniformly weak. Our findings thus establish a comprehensive evidence base against which the efficacy of alternative types of informational interventions may be judged.

## MATERIALS AND METHODS

We coordinated on the design of seven field experiments in six countries that provided information on politician or party performance to voters and assessed the consequences for electoral behavior. One study could not be implemented because of challenges in the field. We collectively preregistered our hypotheses, measures, and the estimating equations used in our meta-analysis (see the MPAP in section S1.1). In the Supplementary Materials, we provide further information on the individual study designs (section S1.2) and describe the information delivered in each study’s common intervention arm (section S1.3). To test our hypotheses about the effects of positive and negative information, we divided subjects into groups, based on whether they would receive good or bad news if exposed to the treatment (see the Supplementary Materials for definitions). In all but one study, preintervention surveys allowed us to define these good and bad news groups based on the individuals’ prior beliefs. We randomly assigned the information treatment to some respondents and not to others within the good and bad news groups. Section S1.4 reports descriptive statistics, while section S1.5 shows that treatment and control groups are statistically balanced on prognostic pretreatment covariates. Section S1.6 reports an ex post simulation that shows our statistical power to detect effects.

### Statistical analysis

In Figs. 2 and 3, we report average treatment effects estimated with block-average differences of means within each of the good and bad news subgroups, without covariate adjustment. The *p*-values calculated by randomization inference can differ from those implied by the model-based CIs, also reported in Figs. 2 and 3, because the randomization tests accurately reflect complexities of the individual designs, such as blocking or clustering of random assignment. For the overall analysis in Figs. 2 and 3, we take as our primary estimand the average of the six study-specific treatment effects. Accordingly, our estimator weighs each study by the inverse of its size. Unweighted analysis produces substantively similar results, as does covariate-adjusted analysis (see the Supplementary Materials for details).

## Supplementary Material

http://advances.sciencemag.org/cgi/content/full/5/7/eaaw2612/DC1

Download PDF

## SUPPLEMENTARY MATERIALS

Supplementary material for this article is available at http://advances.sciencemag.org/cgi/content/full/5/7/eaaw2612/DC1 Section S1. Study design materials and methods Section S2. Primary analysis: Robustness and reliability of results Section S3. Secondary analysis: A Bayesian approach Section S4. Possible explanations for the null findings Section S5. Effects of publicly disseminated information Table S1. Individual study designs. Table S2. Descriptive statistics for sample of good news. Table S3. Descriptive statistics for sample of bad news. Table S4. Balance of covariates. Table S5. Effect of information, conditional on distance between information and priors, on vote choice, and turnout. Table S6. Deviations from MPAP and study PAPs in the meta-analysis. Table S7. Differential attrition. Table S8. Manipulation check: Effect of treatment on correct recollection, pooling good and bad news (unregistered analysis). Table S9. Manipulation check: Absolute difference between posterior and prior beliefs for pooled good and bad news (unregistered analysis). Table S10. Effect of information on perception of importance of politician effort and honesty. Table S11. Effect of information and source credibility on evaluation of politician effort and honesty (unregistered analysis). Table S12. Relationship between evaluation of politician effort and honesty with vote choice (unregistered analysis). Table S13. Effect of bad news on politician backlash. Table S14. Additional hypotheses and results. Table S15. Effect of moderators on incumbent vote choice. Table S16. Effect of information and context heterogeneity on incumbent vote choice. Table S17. Effect of information and electoral competition on vote choice. Table S18. Effect of information and intervention-specific heterogeneity on vote choice. Table S19. Interaction analysis: Effect of good news on incumbent vote choice. Table S20. Interaction analysis: Effect of bad news on incumbent vote choice. Table S21. Private versus public information: Effect of good news on incumbent vote choice. Table S22. Private versus public information: Effect of bad news on incumbent vote choice. Fig. S1. Benin—Graphical representation of provided information. Fig. S2. Brazil—Flyers distributed to voters. Fig. S3. Burkina Faso—Flashcard illustrations of municipal performance indicators. Fig. S4. Mexico—Example of benchmarked leaflet in Ecatepec de Morelos, México. Fig. S5. Uganda 1—Candidate answering questions during a recording session and candidate as seen in video. Fig. S6. Power analysis of minimal detectable effects, computed using Monte Carlo simulation. Fig. S7. Bayesian meta-analysis: Vote choice. Fig. S8. Bayesian meta-analysis: Turnout.
