# Supplementary material for: Voter information campaigns and political accountability: Cumulative findings from a preregistered meta-analysis of coordinated trials
Source: Sci Adv. 2019 Jul 3;5(7):eaaw2612. doi: 10.1126/sciadv.aaw2612 (PMC6609214; doi:10.1126/sciadv.aaw2612)
Supplement: http://advances.sciencemag.org/cgi/content/full/5/7/eaaw2612/DC1 [file supp_5_7_eaaw2612__index.html]

Science Advances | Science AdvancesAAASSearchScience AdvancesMenu

## Supplementary Materials

**This PDF file includes:**

- Section S1. Study design materials and methods
- Section S2. Primary analysis: Robustness and reliability of results
- Section S3. Secondary analysis: A Bayesian approach
- Section S4. Possible explanations for the null findings
- Section S5. Effects of publicly disseminated information
- Table S1. Individual study designs.
- Table S2. Descriptive statistics for sample of good news.
- Table S3. Descriptive statistics for sample of bad news.
- Table S4. Balance of covariates.
- Table S5. Effect of information, conditional on distance between information and priors, on vote choice, and turnout.
- Table S6. Deviations from MPAP and study PAPs in the meta-analysis.
- Table S7. Differential attrition.
- Table S8. Manipulation check: Effect of treatment on correct recollection, pooling good and bad news (unregistered analysis).
- Table S9. Manipulation check: Absolute difference between posterior and prior beliefs for pooled good and bad news (unregistered analysis).
- Table S10. Effect of information on perception of importance of politician effort and honesty.
- Table S11. Effect of information and source credibility on evaluation of politician effort and honesty (unregistered analysis).
- Table S12. Relationship between evaluation of politician effort and honesty with vote choice (unregistered analysis).
- Table S13. Effect of bad news on politician backlash.
- Table S14. Additional hypotheses and results.
- Table S15. Effect of moderators on incumbent vote choice.
- Table S16. Effect of information and context heterogeneity on incumbent vote choice.
- Table S17. Effect of information and electoral competition on vote choice.
- Table S18. Effect of information and intervention-specific heterogeneity on vote choice.
- Table S19. Interaction analysis: Effect of good news on incumbent vote choice.
- Table S20. Interaction analysis: Effect of bad news on incumbent vote choice.
- Table S21. Private versus public information: Effect of good news on incumbent vote choice.
- Table S22. Private versus public information: Effect of bad news on incumbent vote choice.
- Fig. S1. Benin—Graphical representation of provided information.
- Fig. S2. Brazil—Flyers distributed to voters.
- Fig. S3. Burkina Faso—Flashcard illustrations of municipal performance indicators.
- Fig. S4. Mexico—Example of benchmarked leaflet in Ecatepec de Morelos, México.
- Fig. S5. Uganda 1—Candidate answering questions during a recording session and candidate as seen in video.
- Fig. S6. Power analysis of minimal detectable effects, computed using Monte Carlo simulation.
- Fig. S7. Bayesian meta-analysis: Vote choice.
- Fig. S8. Bayesian meta-analysis: Turnout.

Download PDF

**Files in this Data Supplement:**

- Adobe PDF - aaw2612\_SM.pdf
