# Supplementary material for: Voter information campaigns and political accountability: Cumulative findings from a preregistered meta-analysis of coordinated trials
Source: Sci Adv. 2019 Jul 3;5(7):eaaw2612. doi: 10.1126/sciadv.aaw2612 (PMC6609214; doi:10.1126/sciadv.aaw2612)
Supplement: Download PDF [file aaw2612_SM.pdf]

Science Advances
AAAS

advances.sciencemag.org/cgi/content/full/5/7/eaaw2612/DC1

Supplementary Materials for

Voter information campaigns and political accountability: Cumulative findings from a preregistered meta-analysis of coordinated trials

Thad Dunning*, Guy Grossman, Macartan Humphreys, Susan D. Hyde, Craig McIntosh, Gareth Nellis, Claire L. Adida, Eric Arias, Clara Bicalho, Taylor C. Boas, Mark T. Buntaine, Simon Chauchard, Anirvan Chowdhury, Jessica Gottlieb, F. Daniel Hidalgo, Marcus Holmlund, Ryan Jablonski, Eric Kramon, Horacio Larreguy, Malte Lierl, John Marshall, Gwyneth McClendon, Marcus A. Melo, Daniel L. Nielson, Paula M. Pickering, Melina R. Platas, Pablo Querubín, Pia Raffler, Neelanjan Sircar

*Corresponding author. Email: thad.dunning@berkeley.edu

Published 3 July 2019, *Sci. Adv.* **5**, eaaw2612 (2019)
DOI: 10.1126/sciadv.aaw2612

This PDF file includes:

Section S1. Study design materials and methods
Section S2. Primary analysis: Robustness and reliability of results
Section S3. Secondary analysis: A Bayesian approach
Section S4. Possible explanations for the null findings
Section S5. Effects of publicly disseminated information
Table S1. Individual study designs.
Table S2. Descriptive statistics for sample of good news.
Table S3. Descriptive statistics for sample of bad news.
Table S4. Balance of covariates.
Table S5. Effect of information, conditional on distance between information and priors, on vote choice, and turnout.
Table S6. Deviations from MPAP and study PAPs in the meta-analysis.
Table S7. Differential attrition.
Table S8. Manipulation check: Effect of treatment on correct recollection, pooling good and bad news (unregistered analysis).
Table S9. Manipulation check: Absolute difference between posterior and prior beliefs for pooled good and bad news (unregistered analysis).
Table S10. Effect of information on perception of importance of politician effort and honesty.
Table S11. Effect of information and source credibility on evaluation of politician effort and honesty (unregistered analysis).
Table S12. Relationship between evaluation of politician effort and honesty with vote choice (unregistered analysis).
Table S13. Effect of bad news on politician backlash.
Table S14. Additional hypotheses and results.

Table S15. Effect of moderators on incumbent vote choice.
Table S16. Effect of information and context heterogeneity on incumbent vote choice.
Table S17. Effect of information and electoral competition on vote choice.
Table S18. Effect of information and intervention-specific heterogeneity on vote choice.
Table S19. Interaction analysis: Effect of good news on incumbent vote choice.
Table S20. Interaction analysis: Effect of bad news on incumbent vote choice.
Table S21. Private versus public information: Effect of good news on incumbent vote choice.
Table S22. Private versus public information: Effect of bad news on incumbent vote choice.
Fig. S1. Benin—Graphical representation of provided information.
Fig. S2. Brazil—Flyers distributed to voters.
Fig. S3. Burkina Faso—Flashcard illustrations of municipal performance indicators.
Fig. S4. Mexico—Example of benchmarked leaflet in Ecatepec de Morelos, México.
Fig. S5. Uganda 1—Candidate answering questions during a recording session and candidate as seen in video.
Fig. S6. Power analysis of minimal detectable effects, computed using Monte Carlo simulation.
Fig. S7. Bayesian meta-analysis: Vote choice.
Fig. S8. Bayesian meta-analysis: Turnout.

Section S1. Study design and methods

1.1 Meta Pre-Analysis Plan (MPAP)

We first reproduce the meta pre-analysis plan, which was filed on March 9, 2015 at `http://egap.org/registration/736`, prior to the fielding of interventions and before collection of baseline data. Note that we have corrected minor spelling mistakes and made minor editorial changes, including bringing footnotes into the text, to conform with *Science Advances* journal formatting requirements. We otherwise present the MPAP as filed.

Political Information and Electoral Choices: A Meta-Preanalysis Plan (MPAP)

Thad Dunning[#8] Guy Grossman[#8] Macartan Humphreys[#8]
Susan Hyde[#8] Craig McIntosh[#8] Claire Adida[#1] Eric Arias[#2]
Taylor Boas[#4] Mark Buntaine[#7] Sarah Bush[#7] Simon Chauchard[#3]
Jessica Gottlieb[#1] F. Daniel Hidalgo[#4] Marcus E. Holmlund[#5]
Ryan Jablonski[#7] Eric Kramon[#1] Horacio Larreguy[#2] Malte Lierl[#5]
Gwnyeth McClendon[#1] John Marshall,[#2] Dan Nielson[#7]
Melina Platas Izama[#6] Pablo Querubin[#2] Pia Raffler[#6]
Neelanjan Sircar[#3].

March 9, 2015

Abstract

We describe our plan for a meta analysis of a collection of seven studies on the impact of information on voting behavior in developing countries. The seven studies are being conducted simultaneously by seven separate research teams under a single "Metaketa" grant round administered by EGAP and University of California, Berkeley's Center on the Politics of Development. This analysis plan has been produced before launch of any of the seven projects and provides the analysis for the joint assessment of results from the studies. Individual studies have separate pre-analysis plans with greater detail, registered prior to the launch of each study.

Author annotations are: # 1 Benin study, #2 Mexico study, #3 India study, #4 Brazil study, #5 Burkina Faso study, #6 Uganda 1 study, #7 Uganda 2 study, #8 the *Metaketa* committee. We have many people to thank for generous thoughts and comments on this project including Jaclyn Leaver, Abigail Long, Betsy Paluck, Ryan Moore, Ana de la O, Don Green, Richard Sedlmayr, and participants at EGAP 13. The Metaketa is funded by an anonymous donor.

Contents

1 Introduction **1**

2 Interventions and Motivation **1**
2.1 Primary Intervention Arm . . . 1
2.2 Secondary Intervention Arm . . . 2
2.3 Additional variations . . . 3

3 Hypotheses **4**
3.1 Primary Hypotheses . . . 4
3.2 Hypotheses on Secondary Outcomes . . . 4
3.3 Hypotheses on Intermediate Outcomes . . . 5
3.4 Hypotheses on Substitution Effects . . . 5
3.5 Context Specific Heterogeneous Effects . . . 5
3.6 Intervention Specific Heterogeneous Effects . . . 6

4 Measurement **6**
4.1 Outcome measures . . . 6
4.1.1 Vote choice . . . 6
4.1.2 Turnout . . . 7
4.1.3 Intermediate outcomes . . . 7
4.2 Priors on Treatment Information . . . 8
4.3 Controls and Moderators . . . 8
4.3.1 Individual level items . . . 8
4.3.2 Treatment level items . . . 9
4.3.3 Election (race) level features . . . 10
4.3.4 Country Level data . . . 10
4.3.5 Manipulation Checks . . . 10

5 Analysis details **10**
5.1 Main Analysis . . . 11
5.2 Analysis of Heterogeneous Effects . . . 12
5.3 Adjustment for multiple comparisons . . . 12
5.4 Contingencies . . . 14
5.4.1 Non-Compliance . . . 14
5.4.2 Attrition . . . 14
5.4.3 Missing data on control variables . . . 14

6 Additional (secondary) analysis **14**
6.1 Randomization checks and balance tests . . . 14
6.2 Disaggregated analyses . . . 14
6.3 Controls . . . 14
6.4 Possible additional analysis of official data . . . 15
6.5 Bayesian hierarchical analysis model . . . 15
6.6 Exploratory analysis . . . 16
6.7 Learning about learning . . . 16

7 Ethics **17**

8 Caveats **17**

1 Introduction

In this document we describe the research and analysis strategy for an EGAP "*Metaketa*" on information and accountability. *Metaketas* are integrated research programs in which multiple teams of researchers work on coordinated projects in parallel to generate generalizable answers to major questions of scholarly and policy importance. The core pillars of the Metaketa approach are:

1. **Major themes**: Metaketas focus on major questions of scholarly and policy relevance with a focus on consolidation of knowledge rather than on innovation.

2. **Strong designs:** all studies employ randomized interventions to identify causal effects.

3. **Collaboration and competition:** teams work on parallel coordinated projects and collaborate on design and on both measurement and estimation strategies in order to allow for informed comparisons across study contexts.

4. **Comparable interventions and measures:** differences in findings should be attributable primarily to contextual factors and not to differences in research design or measurement.

5. **Analytic transparency:** all studies share a commitment to analytic transparency including design registration, open and replicable data and materials, and third-party analysis prior to publication.

6. **Formal synthesis:** aggregation of results of the studies is achieved though pre-specified meta-analysis and via integrated publication platform to avoid publication bias.

The Information and Accountability Metaketa was launched in Fall 2013 and will run until Spring 2018. Its key objective is to implement a series of integrated experimental projects that assess the role of information in promoting political accountability in developing countries. This Metaketa is being administered by the Center on the Politics of Development at the University of California, Berkeley. This first registration document (dated: March 15, 2015) has been posted publicly to the EGAP registry prior to the administration of treatment in any of seven projects taking part in this Metaketa.

2 Interventions and Motivation

Civil society groups and social scientists commonly emphasize the need for high quality public information on the performance of politicians as an informed electorate is at the heart of liberal theories of democratic practice. The extent to which performance information in effect make a difference in institutionally weak environments is, however, an open question. Specifically when does such information lead to the rewarding of good performance candidates at the polls and when are voting decisions dominated by nonperformance criteria such as ethnic ties and clientelistic relations?
The studies in this project address the above questions by examining a set of interventions that provide subjects with information about key actions of incumbent political representatives. We assess the effects of providing this information on vote choice and turnout, given prior information available to voters.

2.1 Primary Intervention Arm

Each of the seven projects has at least two treatment arms. The first arm is an informational intervention focused explicitly on the performance of politicians. While the specific political office (e.g., mayor or member of parliament), the type of performance information provided, and the medium

for communicating the information vary somewhat across studies, the interventions are designed to be as similar as possible to each other; they are also similar to several previous informational interventions in research on political accountability. Most importantly, each intervention is designed to allow voters to update their beliefs about the performance of the politicians positively or negatively in light of the information. The extent to which such updating actually takes place will play a key role in comparing the impact of the performance information across contexts.
A very brief description of the primary informational treatment, T1, in each study is included in Table 1 below. We summarize the interventions here; for more details, see the pre-analysis plans for each individual study.

- In **Benin**, researchers provide information to respondents on indices of **legislative performance** of deputies in the National Assembly. Videos featuring bar graphs highlight the performance of the legislator responsible for each commune and present this information relative to other legislators in the department (a local average) and the country (national average).
- In **Mexico**, researchers provide information in advance of municipal elections on **corruption** (measured as the share of total resources that are used in an unauthorized manner) or on **misuse of public funds** (the share of resources that have benefited non-poor individuals from funds that are explicitly earmarked to poor constituents).
- In **India**, researchers provide information on **criminal backgrounds of candidates** in state assembly races. Publicly available information, culled from India's Election Commission, will be disseminated in a door-to-door campaign across 18 randomly selected polling booths within 25 electoral constituencies in the Indian state of Bihar.
- In **Brazil**, researchers will distribute information about general government **corruption** in mayoral races. In partnership with the Accounts Court in the northeastern state of Pernambuco, the research team will provide voters with information on incumbent malfeasance via report cards and oral communication, drawing on publicly available data from annual auditing reports.
- In **Burkina Faso**, researchers provide information on the performance of municipal governments with respect to national targets for public service delivery. After pilot tests, it will be determined whether this information will be presented in the form of relative performance rankings of the municipalities within a region, or in the form of scores that indicate a municipality's performance relative to normative targets.
- In **Uganda (study 1)**, researchers provide information on **service delivery in Parliamentary constituencies** using scorecards.
- In **Uganda (study 2)**, researchers will use text-messaging (SMS) to provide information on **service delivery** in district government races. Specifically the researchers will disseminate information on local government budget allocations, as well as comparative quality of public services (roads, water supply, and solid waste).

2.2 Secondary Intervention Arm

The studies include second arms that test conditions under which the provision of information might be more or less effective. As part of the second arm, studies assess the effects of variation in the *message content* (absolute or relative information), the *type of messenger* (surveyor vs. community elites), and *delivery method* (providing information collectively vs. individually to groups of voters). Many studies compare a *public* treatment which may generate *common knowledge* of the intervention to a private baseline. We refer to these secondary interventions as T2:

- In **Benin**, researchers use a 2×2 factorial design plus pure control. One dimension of the factorial design concerns whether the information is provided in a *public* or *private* fashion. In the public condition, the informational video will be screened in a public location; a random sample of villagers will be invited to the film screening. In the private condition, the same video will be shown to randomly sampled individual in households in one-to-one interactions. The other dimension crosses the presence or absence of a *civics message* highlighting the implications of poor legislator performance for voter welfare.

- In **Mexico**, researchers use a 2×2 factorial design plus control. Similarly to the Benin study, one treatment group will receive information about municipal-level corruption and misuse of funds only privately (via fliers) whereas another will receive this information in a public manner (using cars with megaphones). The other cross-cutting dimension concerns whether or not citizens also receive benchmark information about the state average.

- In **India**, this study examines the causal effect of the information "messenger". In one treatment group, surveyors will distribute a flyer and summarize the information included in the flyer in face-to-face interactions. In the second treatment group, locally influential individuals will be contracted to disseminate the exact same information in a similar manner.

- The **Brazil** study explores the effect of varying the saliency of the information communicated to voters. Specifically, for the alternative arm, the researchers will provide information on mayoral compliance with a highly salient crop insurance program, which allows testing for the importance of providing information on policies directly relevant to voters' lives.

- In **Burkina Faso**, the alternative arm includes a personal invitation to a municipal council meeting. Here first-hand experience with the municipal decision process is expected to make the political information disseminated as part of the main (common) arm more salient to citizens.

- In **Uganda (study 1)**, researchers will provide information via screenings of structured debates of parliamentary candidates. The researchers plan to exploit an additional source of variation: intra- vs. inter-party competition (i.e. primaries of the ruling party vs general election). The idea is to explore whether performance information is more likely to have a bite in primary settings when the impact of partisanship on vote choice is minimized.

- In **Uganda (study 2)**, researchers will vary the saturation of the level of information.

Because treatments in the second arm differ across studies by design, we will not conduct pooled analysis or formal comparison of the effects of many of these treatments. However, our final report and publications will present estimates of the effects of the second arm in each study, both in absolute terms and relative to the first arm in each project. In addition, we will compare the pooled effects of *private* vs. *public* treatments, as a way to assess whether the generation of common knowledge may strengthen the effects of informational interventions. These analyses may provide important hypotheses for further studies to assess rigorously, for example, through Metaketas in which promising secondary arms in our set of studies are tested as primary (common) arms.

2.3 Additional variations

We inform respondents in surveys (including those assigned to control groups) that they may be provided with information on candidate quality, and we seek consent to participate. While this enhances subject autonomy, it also risks creating Hawthorne-type biases. To assess this possibility, a set of studies will also employ a variation, T3, that randomly varies the consent script among control units (though consent for measurement is sought in all cases).

MPAP Table 1: Primary Informational Intervention Across Projects

Project	Title	PIs	Information on...	Method
**Benin**	Can Common Knowledge Improve Common Goods?	Adida, Gottlieb, Kramon, & McClendon	Legislative performance of **deputies in the National Assembly**	Legislator performance info provided publicly or privately & a civics message
**Mexico**	Common Knowledge, Relative Performance & Political Accountability	Larreguy, Querubin, Arias, & Marshall	Corruption & the misuse of public funds by **local government officials**	Leaflets distr. door-to-door complemented w/cars with loudspeakers drawing attention to the provided leaflets
**India**	Using Local Networks to Increase Accountability	Chauchard & Sircar	Financial crimes by **members of the state assembly**	Door-to-door campaigns vs. public rallies
**Brazil**	Accountability & Incumbent Performance in the Brazilian Northeast	Hidalgo, Boas, & Melo	Performance gathered from audit reports of the **local government**	Report cards & an oral message
**Burkina Faso**	Citizens at the Council	Lierl & Holmlund	Service delivery by the **municipal government**	Scorecard & participation in local council meetings
**Uganda I**	Information & Accountability in Primary & General Elections	Raffler & Platas Izama	Service delivery by the **local government**	Recorded candidate statements viewed publicly & privately
**Uganda II**	Repairing Information Underload	Nielson, Buntaine, Bush, Pickering & Jablonski	Service delivery by the **local government**	Information sent by SMS to randomly sampled households.

3 Hypotheses

We now lay out six *families* of hypotheses which will be tested across the seven studies.

3.1 Primary Hypotheses

We have two closely related primary hypotheses:

H1a Positive information increases voter support for politicians (subgroup effect).

H1b Negative information decreases voter support for politicians (subgroup effect).

We define positive information, i.e."good news" and negative information i.e. "bad news" in subsection 5.1.

3.2 Hypotheses on Secondary Outcomes

A secondary hypotheses relates to overall participation. Theoretical work suggests that greater information should increase turnout, whether it is good or bad; yet recent experimental evidence finds that information that highlights corruption may reduce engagement with electoral processes. We state distinct hypotheses on turnout as a function of information content though we highlight that our interest is in estimating the relation, whether it is positive, or negative, or context dependent.

H2a Bad news decreases voter turnout.

H2b Good news increases voter turnout.

3.3 Hypotheses on Intermediate Outcomes

We also focus on first-stage relations between treatment and intermediate outcomes. These outcomes could be conceived of as *mediators* that link treatments to our primary and secondary outcomes (vote choice and turnout). However, it is possible not only that beliefs shape behaviors but also that behaviors shape beliefs. We thus do not take a strong position on whether these outcomes are necessarily channels through which treatment affects our primary and secondary outcomes. We also analyze mechanisms by conducting implicit mediation analysis (Gerber and Green 2012), in which we use the variation in treatments across primary and secondary interventions within studies (see H13-H15).

H3 Positive (negative) information increases (decreases) voter beliefs in candidate integrity.

H4 Positive (negative) information increases (decreases) voter beliefs that candidate is hardworking.

H5 Politicians mount campaigns to respond to negative information.

3.4 Hypotheses on Substitution Effects

We expect that information will operate on vote choice in part by reducing the weight voters place on ethnicity, co-partisanship, and clientelistic relations. Thus for example we expect good news to reduce the bias for voting against non-coethnic outgroup candidates and bad news to reduce the bias for voting for coethnic candidates. However, even though information may reduce the weight voters place on these relations, we expect that information has more positive effects for voters that do not share ethnic, partisan, or clientelist ties with candidates.

H6 Information effects are more positive for voters that do not share *ethnic identities*. (This hypothesis is not relevant for all projects, e.g. Mexico and Brazil; see measurement section.)

H7 Information effects are more positive for voters with weaker *partisan identities*.

H8 Information effects are more positive for voters who have not received *clientelistic* benefits from any candidate.

While substitution effects and other heterogeneous effects are important, we note that a causal interpretation of these heterogeneous effects is not justified by the experimental design. We do not manipulate the conditioning covariates in our experiments, and we lack an identification strategy that would allow us to make strong causal claims about the effects of these variables.

3.5 Context Specific Heterogeneous Effects

H6-H8, though related to a logic of mechanisms, are analyzed here in terms of heterogeneous effects. Two other sets of heterogeneous effects are also examined. The first set relates to the electoral environment and reflects expectations that new information will have a bigger impact in informationally poor environments and in settings where votes count—ie where fraud is low and chances of votes making a difference are greater.

H9 Informational effects are stronger in informationally weak environments.

H10 Informational effects are stronger in more competitive elections.

H11 Informational effects are stronger in settings in which elections are believed to be free and fair.

3.6 Intervention Specific Heterogeneous Effects

A final set of heterogeneous effects analyses relate to the design of the interventions, which differ in part across study, though some of the differences may also have local granularity.

H12 Information effects—both positive and negative—are stronger when the gap between voters' prior beliefs about candidates and the information provided is larger.

H13 Informational effects are stronger the more the information relates directly to individual welfare.

H14 Informational effects are stronger the more reliable and credible is the information *source*.

H15 Informational effects are stronger when information is provided in *public settings*.

H16 Informational effects are not driven by Hawthorne effects.

4 Measurement

4.1 Outcome measures

This section outlines core measures that are common to all project teams. Most project teams will measure additional outcomes as specified in individual pre-analysis plans.

4.1.1 Vote choice

M1 VOTECHOICE. The primary outcome is *individual level vote choice*. The measure takes a value of 1 if the constituentt voted for the *incumbent* (or the incumbent's party when no incumbent is up for reelection) and 0 if she did not (whether or not she actually voted). Teams may ask the question about vote choice in different ways, seeking to maximize reliability of the measure in each context (sample question below). All teams asking the question in face-to-face will ask sampled respondents to place a vote in a ballot box.

- When possible the measurement of vote choice should take place before official results are announced.
- This should take place in private when possible.
- Only the researcher has an ability to connect between a code on the envelope and the identity of respondents.

When PIs collect individual-level vote choice remotely via telephone or USSD/SMS, the following principles apply:

- Data collection needs to take place before official results are announced.
- Respondents should be contacted by automated voice system or USSD with random question order and random response choice to prevent sample-level reconstruction of the data.
- PIs need positive consent in the case where they cannot guarantee encryption of messages / voice response. Encryption is dependent on particular mobile service networks.

Sample question:

- `For which [candidate/party] did you vote for [MP/Mayor/Councilor] in the most recent [type of election] elections.`

M2 OFFICIALVOTE. Official vote choice data. Whenever possible, teams will assemble *polling station-level vote choice* outcomes using official electoral commission data.

4.1.2 Turnout

M3 TURNOUT.: Teams that measure individual-level treatment effects will measure individual turnout. Measures will be employed in the following order:

(a) Use individual-level turnout data from the official electoral commission, where available.

(b) Use direct survey responses, even as surveys tend to inflate turnout due to social desirability bias. Confirmations such as ink marks should be sought whenever possible.

M4 GROUPTURNOUT: Teams that measure group-level treatment effects will measure turnout at the level used for randomization when possible.

(a) First best is using official electoral commission data at the polling station level (or other level, if randomization is at that level), if possible.

(b) Second best is to use the share of sampled respondents that have voted. The key here is to go back to villages/municipalities/localities immediately after the election and ask to verify vote through official marking (in ID, ink, etc.)

4.1.3 Intermediate outcomes

These intermediate outcomes are likely to be affected by treatments and can offer insight into the mechanisms at play—and thus may be mediators. All studies will measure a core set of beliefs about attributes of incumbents, specified below. Project teams may, however, measure additional mediators as specified in individual pre-analysis plans.

M5 EFFORT.: Evaluation of the extent to which a politician is hardworking/provides effort.

– `In your opinion, does [INCUMBENT] make much more, a little more, a little less or much less effort to get things done than other deputies in this [Department]?`

M6 HONESTY.: Evaluation of the extent to which a politician is honest.

– `How surprised would you be to hear from a credible source about corruption involving your [MP/Mayor/Councilor]? Would you say you would be (1) Very surprised (2) Somewhat surprised (3) Not too surprised (4) Not surprised at all`

M7 CRITERIA. Did the respondent change the criteria they used to evaluate candidate? (endline)

– `What was most important to you when deciding which [candidate/party] to support in the [Type] election? [Enumerator codes each of the following elements of answers; may be asked as a closed-ended question if necessary, e.g., for Uganda 2 survey]:`

1. `Identity (ethnicity; group representation)`
2. `Personal benefits targeted at voter or their family`
3. `Local benefits`
4. `National or policy contributions`
5. `How hardworking the politician is (effort)`
6. `Character of politician (integrity)`
7. `Endorsements by others (leaders; family members).`

M8 BACKLASH. Did politicians respond to information provided at cluster level? Cluster average of: (endline)

– `In the week before the election did you hear of [incumbent] or someone from their party making statements about [dimension of information provided to treated groups]?`

4.2 Priors on Treatment Information

M9 PRIORS (P) All groups will gather information on voter priors at baseline (in both treatment and control groups) with respect to the information that will be provided. (One project team (Mexico) will not conduct a baseline survey due to prohibitive costs. This team will instead gather aggregate information at the precinct level (the level of treatment assignment) on priors in the control group at endline.) Where possible, this will be gathered on the same scale as the information that will eventually be provided.

Example from Benin: `Consider [NAME OF REP], does she/he participate in plenary sessions of the National Assembly much more, a little more, a little less or much less than other deputies in this Department? (1) Much more; (2) A little more; (3) A little less; (4) Much less.`

M10 GOODNEWS. An indicator of "good news" is generated based on M9 and the information provided to treatment groups (see subsection 5.1).

M11 CERTAIN. A measure of how certain voters are about their prior opinions in M9:

- `How certain are you about your response to this question? (1) Very certain; (2) Certain; (3) Not certain; (4) Very uncertain.`

M12 CLUSTERPRIORS. Group priors are given by the cluster level of average priors as measured by M9.

4.3 Controls and Moderators

Moderators are contextual factors that are not affected by the treatments, but that might be responsible for heterogeneous treatment effects. A core set of measures will be harmonized across studies. Project teams might measure additional controls and moderators as specified in individual pre-analysis plans.

4.3.1 Individual level items

M13 GENDER (baseline).

M14 AGE (baseline): `year of birth`

M15 COETHNIC (baseline):

- `Thinking of the [candidate for MP/Mayor/Councilor], would you say that [you come from the same community/share the same ethnic group/share the same race] as this candidate?`

This is a subjective measure of co-ethnicity. (More objective measures of co-ethnicity are challenging to develop in all study contexts, especially in Mexico and Brazil.) Teams may wish to develop additional study-specific measures appropriate to each context.

M16 COGENDER. Whether the individual is of the same gender as the candidate *about which information will be provided to the treatment group(s)* (baseline)

M17 EDUCATION : `number of years of education` (baseline)

M18 WEALTH (baseline)

- `In general, how do you rate your living conditions compared to those of other [Brazilians/Mexicans/Indians/Beninois/Burkinabés/Ugandans]? Would you say they are much worse, worse, the same, better, or much better?`

M19 PARTISAN (baseline).

- `On this scale of one to seven, where seven means you are very attached to [`*`INCUMBENT'S PARTY`*`], and one means you are not very attached to [`*`INCUMBENT'S PARTY`*`], what degree of attachment do you feel for [`*`INCUMBENT'S PARTY`*`]?`

M20 VOTED (baseline):

- `Did you vote in the last [...] elections?`

M21 SUPPORTED (baseline)

- `Did you support the incumbent in the last [...] elections?`

M22 CLIENTELISM (baseline)

- `How likely is it that the incumbent, or someone from their party, will offer something, like food, or a gift, or money, in return for votes in the upcoming election (1) Not at all likely (2) Not very likely (3) Somewhat likely (4) Very likely`

4.3.2 Treatment level items

M23 SALIENT (baseline): Measure of the extent to which information *provided in the primary treatment arm* relates to welfare (baseline).

`I am going to read you a list of activities in which your [REP] could be involved. Suppose you could receive information about one of these things. I'd like to ask you to tell me about which of these activities you would most like to receive information:`

(a) `How well the politician performs his/her duties in the [national legislature], for example, attendance in plenary sessions and council or committee meetings`
(b) `Whether the politician has been engaged in corruption`
(c) `Whether the politician has been accused of committing a crime`
(d) `Whether the politician is effective at delivering services and bringing benefits to this community`

`...Now, thinking of the previous question, please tell me a second activity about which you would like to receive information about your [MP/Mayor/Councilor] [read three options not previously chosen]`
`...Now, thinking of the previous question, please tell me a third activity about which you would like to receive information about your [MP/Mayor/Councilor] [read two options not previously chosen]`

M24 SOURCE. Credibility of the information source:

`Suppose that you received information about a politician, for example, information about how he or she had performed in office. Which of the following sources would you trust the most [second most; third most] for that information? [READ OPTIONS]:`

(a) `Local politician`
(b) `Flyer or pamphlet from an NGO`
(c) `A person conducting a survey`
(d) `An influential member of your community`
(e) `In a debate between candidates`
(f) `Other`

4.3.3 Election (race) level features

M25 COMPETITIVENESS. This measure will vary across systems.

- For candidates elected through single-member/first-past-the-post elections, this is 1 minus the margin of victory of the incumbent 1-(vote share - vote share of runner up) (historical data from the electoral commission).
- For proportional representation (closed list) systems, a candidate ranked in position k of a party that received m seats out of n, is accorded competitiveness score of $1 - (1 + m - k)/n$. Thus individuals positioned 1,2,3 in a party that received 3 out of 7 seats have competitiveness scores 4/7, 5/7, 6/7 respectively.
- For proportional representation (open list) systems, this is the difference in raw votes of the incumbent and the vote share of the candidate who received the largest number of votes and did not receive a seat

A general measure of free and fairness will be made by averaging standardized versions of the following two measures:

M26 SECRETBALLOT: Voter confidence in the secret ballot (baseline)

- `How likely do you think it is that powerful people can find out how you vote, even though there is supposed to be a secret ballot in this country? (1) Not at all likely (2) Not very likely (3) Somewhat likely (4) Very likely`

M27 FREEANDFAIR: Voter beliefs that the election will be free and fair in constituency (baseline)

- `How likely do you think it is that the counting of votes in this election will be fair (1) Not at all likely (2) Not very likely (3) Somewhat likely (4) Very likely`

4.3.4 Country Level data

M28 FREEPRESS. Freedom House measure of freedom of the press

M29 DEMOC. Polity measure of democratic strength

4.3.5 Manipulation Checks

Manipulation checks data is also gathered which can be used to assess whether treatment groups absorbed the treatment (i.e., did the individual understand the information?); whether control groups learned more about representatives between baseline and the election; and whether there was informational spillovers between treated and control units.

M30 CHECK At endline, data should be gathered from treatment and control groups about the performance of representatives using the same approach as used for Measure M9. (Here we recognize that voters could have absorbed the information and yet posteriors over candidates on the dimension of the information may not have budged—perhaps because voters filter the information through partisan lenses.)

5 Analysis details

In this section we describe the primary empirical strategy that will be used to test the above set of hypotheses across studies.

The most straightforward way to combine results across the seven studies pools units into one large study group and estimates treatment effects, as one would do in a large experiment in which treatment assignment is blocked. For this analysis we proceed as if blocking is implemented at the country level.

From one perspective, this approach involves weak assumptions. The study group in the large experiment is not conceived as a random sample from a larger population. This follows from the design of the studies: in most of the seven projects, individuals in the study groups are not themselves random samples, and the study sites (countries and locations within countries) are also not random draws from a well defined population of possible sites. From another perspective, pooling does imply that we can treat interventions and outcome measures as sufficiently comparable that an overall average treatment effect (say, the effect on vote choice of exposure to "good news") is meaningful. Creating such comparability is the goal of the *Metaketa* initiative, but in practice the information that is provided in different projects differs quite substantially, even when focusing explicitly the primary information arm. We account for this heterogeneity partially by formally examining the effects of heterogeneity in our analysis.

5.1 Main Analysis

Since expected effects derive from *new* information rather than *any* information, the core estimates need to take account of both the content of the information and prior beliefs.

Let P_{ij} denote the prior beliefs of voter i regarding some politically relevant attribute of politician j and let Q_j denote the information provided to the treatment group about politician j on that attribute, *measured on the same scale*. Let $\hat{Q}_j$ denote the median value of Q_j in a polity (or, for teams using local comparison groups, the median in the relevant comparison group).

Define L^+ as the set of treatment subjects for whom $Q_j > P_{ij}$ or $Q_j = P_{ij}$ and $Q_j \geq \hat{Q}_j$. These are subjects that receive good news — either the information provided exceeds priors or the information confirms positive priors. Let L^- denote the remaining subjects. Let N^+_{ij} denote the difference $Q_j - P_{ij}$, defined for all subjects in L^+ and standardized by the mean and standard deviation of $Q_j - P_{ij}$ in the L^+ group in each country (or relevant locality). N^+_{ij} is therefore a standardized measure of "good news" with mean 0 and standard deviation of 1. Let N^-_{ij} denote the same quantity but for all subjects receiving bad news.

Then the two core estimating equations are

$$E(Y_{ij}|i \in L^+) = \beta_0 + \beta_1 N^+_{ij} + \beta_2 T_i + \beta_3 T_i N^+_{ij} + \sum_{j=1}^{k} (\nu_k Z^k_i + \psi_k Z^k_i T_i) \quad (1)$$

$$E(Y_{ij}|i \in L^-) = \gamma_0 + \gamma_1 N^-_{ij} + \gamma_2 T_i + \gamma_3 T_i N^-_{ij} + \sum_{j=1}^{k} (\nu_k Z^k_i + \psi_k Z^k_i T_i) \quad (2)$$

where $Z_1, Z_2, ..., Z_k$ are prespecified covariates, also standardized to have a 0 mean.

Here β_2 is the *average treatment effect* of information for all voters receiving good news; γ_2 is the *average treatment effect* of information for all voters receiving bad news. Recall that according to H1a and H1b we expect $\beta_2 > 0$ and $\gamma_2 < 0$. Note that models 1 and 2 assume that potential outcomes (e.g. vote choice or turnout after good news, bad news, or no news) are fixed and may differ from individual to individual; the only random element in the above models is assignment to the treatment condition T_i (given priors, which by definition are determined before treatment assignment).

In addition to reporting these as our primary results we will report the results for the analogous specification without covariates. We will also report the mean value of Y_{ij} by treatment condition for both sets of individuals (those in L^+ and those in L^-), i.e., without conditioning on N^+_{ij} or N^-_{ij}.

Estimation is conducted using OLS, clustering standard errors on politicians (j) and adding fixed effects for constituencies. If treatment assignment is blocked within projects, and treatment assignment probabilities vary across blocks, analysis will account for the blocking, e.g. by the weighting of block-specific effects (or fixed effects for blocks when appropriate). For analysis of aggregate data with clustered assignment, variables are aggregated to their cluster means (where cluster is the level of treatment assignment) or standard errors are clustered at this level. If no uniform weights are used, inverse propensity weights will be employed.

5.2 Analysis of Heterogeneous Effects

Following from the main estimating equations, for a covariate X_{ij} the heterogeneous effect of positive and negative information will be estimated through interaction analysis. Note that we again do not pool since we expect heterogeneous effects to work differently for good news and bad news, as is the case if a covariate is associated with stronger or weaker effects.

$$\text{E}(\text{Y}_{ij}|i \in L^+) = \beta_0+\beta_1 N^+_{ij}+\beta_2 T_i+\beta_3 T_i N^+_{ij}+\beta_4 X_i+\beta_5 T_i X_i+\sum_{j=1}^{k}(\nu_k Z_i^k+\psi_k Z_i^k T_i) eq.het1 \quad (3)$$
$$\text{E}(\text{Y}_{ij}|i \in L^-) = \gamma_0 + \gamma_1 N^-_{ij} + \gamma_2 T_i + \gamma_3 T_i N^-_{ij} + \gamma_4 X_i + \gamma_5 T_i X_i + \sum_{j=1}^{k}(\nu_k Z_i^k + \psi_k Z_i^k T_i) \quad (4)$$

Where X is the variable of interest (which we assume is not included in the set of other covariates Z). The heterogeneous effects of the impact of positive information, for average news levels, are given by β_5 and the heterogeneous effects of negative information are given by γ_5. Note that we do not include a triple interaction between T, X and N^+/N^- in these analyses.
For H12 we can combine data and estimate more simply:

$$E(Y_{ij}) = \delta_0 + \delta_1(Q_j - P_{ij}) + \delta_2 T_i + \delta_3 T_i(Q_j - P_{ij}) \quad (5)$$

Under H12 we expect $\delta_3 > 0$. Note that our measures of $Q_j - P_{ij}$ are largely ordinal not interval; and estimating a linear marginal effect of the gap may not be meaningful if the marginal effect is not in fact linear. Perhaps more importantly, we do not manipulate priors in our experiments, and we lack an identification strategy that would allow us to make strong causal claims about the effects of such a gap. Such caveats should be born in mind, yet we believe it is valuable to assess H12 with the tools at our disposal.
The mapping between hypotheses (section 3) and measures (section 4) is outlined in Table 2.

Where CONTROLS are:

- for individual level specifications: {M14, M15, M16, M17, M18, M19, M20, M21, M22, M26, M27}
- for cluster level specifications: averages of {M15, M17, M18, M19, M20, M21, M22, M26, M27}

5.3 Adjustment for multiple comparisons

We handle multiple comparisons concerns in two ways.
First note that most tests are conducted using pairs of analyses—e.g. the (positive) effect of good news on voting and the (negative) effect of bad news. For each of these pairs of analyses, in addition to the simple p values reported for each regression, we will calculate a p value for the *pair* of regressions which will be given by the probability that *both* the coefficients would be as large (in absolute value) as they are under the sharp null of no effect of exposure to information (good or bad) for any unit. (We calculate this p value using randomization inference. Let $f(b)$ denote a bivariate distribution of coefficients b_1, b_2 generated under the sharp null, and let $b^* = (b_1^*, b_2^*)$

MPAP Table 2: Specifications, Hypotheses and Measures

Family	#	Abbreviated Hypothesis	Y	X	Interact'n	Controls	Subset	Spec'n
Primary	H1a	Good news effects	M1	T1		✠	M10=1	Eq1
(1)	H1b	Bad news effects	M1	T1		✠	M10=0	Eq2
Secondary	H2a	Turnout (Good news)	M3	T1		✠	M10=1	Eq 1
(2)	H2b	Turnout (Bad news)	M3	T1		✠	M10=0	Eq 2
	H4	Candidate effort	M5	T1		✓	M10=1	Eq1
	H4	Candidate effort	M5	T1		✓	M10=0	Eq2
Mediators	H3	Candidate integrity	M6	T1		✓	M10=1	Eq1
(3)	H3	Candidate integrity	M6	T1		✓	M10=0	Eq2
	H5	Candidate responses	M8	T1		✓	M10=0	Eq2
Substitution	H6	Non coethnics	M1	T1	M15	✓	M10=1	Eq3
(4)	H6	Non coethnics	M1	T1	M15	✓	M10=0	Eq4
	H7	Partisanship	M1	T1	M19	✓	M10=1	Eq3
	H7	Partisanship	M1	T1	M19	✓	M10=0	Eq4
	H8	Clientelism	M1	T1	M22	✓	M10=1	Eq3
	H8	Clientelism	M1	T1	M22	✓	M10=0	Eq4
Context	H9	Informational environment	M1	T1	M11	✓	M10=1	Eq3
(5)	H9	Informational environment	M1	T1	M11	✓	M10=0	Eq4
	H10	Competitive elections	M1	T1	M25	✓	M10=1	Eq3
	H10	Competitive elections	M1	T1	M25	✓	M10=0	Eq4
	H11	Free and fair elections	M1	T1	M26+M27	✓	M10=1	Eq3
	H11	Free and fair elections	M1	T1	M26+M27	✓	M10=0	Eq4
Design	H12	Information content	M1	T1		✓	All	Eq5
(6)	H13	Information welfare relevant	M1	T1	M23	✓	M10=1	Eq3
	H13	Information welfare relevant	M1	T1	M23	✓	M10=0	Eq4
	H14	Credible Information	M1	T1	M24	✓	M10=1	Eq3
	H14	Credible Information	M1	T1	M24	✓	M10=0	Eq4
	H15	Public Channels	M1	T1	T2	✓	M10=1	Eq3
	H15	Public Channels	M1	T1	T2	✓	M10=0	Eq4
	H16	Hawthorne	M1	T1	T3	✓	M10=1	Eq3
	H16	Hawthorne	M1	T1	T3	✓	M10=0	Eq4

Here, ✠indicates that we will present results with and without controls; see subsections 5.1 and 5.3.

denote the estimated coefficients. Then the p value of interest is given by $\int \mathbb{1}(\min(|b|) \geq \min(|b^*|)) \times \mathbb{1}(\max(|b|) \geq \max(|b^*|)) f(b) db$, where $\mathbb{1}$ is an indicator function.)

Second, for each of our six families of hypothesis, we will present tests using both nominal p-values and tests that employ a false discovery rate (FDR) correction to control the Type-1 error rate. We will control the FDR at level 0.05. Thus, for a given randomization with m (null) hypotheses and m associated p-values, we order the realized nominal p-values from smallest to largest, $p_{(1)} \leq p_{(2)} \leq \ldots \leq p_{(m)}$. Let

$$k \text{ be the largest } i \text{ for which } p_{(i)} \leq \frac{i}{m} 0.05$$

Then, we reject all $H_{(i)}$ for $i = 1, 2, \ldots, k$, where $H_{(i)}$ is the null hypothesis corresponding to $p_{(i)}$. Note that FDR corrections will be implemented using the estimated p values from pairs of tests. Thus for example if in a family there are three pairs of tests, then the FDR correction will be applied using three p values, one extracted from each pair.

We consider as families of tests those outlined in Table 2. For example, for the primary hypotheses and outcomes, we consider good news effects and bad news effects on vote choice (with and without controls); for the primary hypotheses and secondary outcomes, we consider good news and bad news effects on turnout (with and without controls).

5.4 Contingencies

5.4.1 Non-Compliance

Studies will analyze subjects according to their treatment assignation under the intended design, and the primary analysis will ignore non-compliance or failure to treat due to logistical mishaps.

5.4.2 Attrition

If there is attrition for entire blocks containing four or more treatment and control units (for example if entire studies fail to complete or if regions within countries become inaccessible) these blocks will be dropped from analysis without adjustment unless there is substantive reason to believe the attrition is due to treatment status.

Studies will test for two forms of attrition. First, are levels of attrition different across treatment and control groups? Second, are the *correlates of attrition* differential between the treatment and control? The former test will be conducted by comparing mean attrition in treatment and control groups, and reporting t-test statistics. The second test will be conducted regressing an attrition indicator on the interactions of treatment and the core baseline control measures specified above and reporting the F-statistic for all of the interacted variables.

Data from studies that find no evidence for problematic attrition from these two tests will be analyzed ignoring attrition.

If differential attrition is detected, Lee bounding techniques will be used to provide estimates of the magnitude of bias that could have resulted from differential attrition, from problematic studies, as well as testing whether the core findings of the study are robust to the observed rate of differential attrition.

5.4.3 Missing data on control variables

If there is missing data on control variables, missing data will be imputed using block mean values for the lowest block for which data is available.

6 Additional (secondary) analysis

In addition to the core analyses descibed above we will undertake a set of secondary analyses.

6.1 Randomization checks and balance tests

Using the full set of baseline covariates described in this document we will report study-by-study F statistics for the hypothesis that all covariates are orthogonal to treatment. In addition we will report balance for all covariates in terms of the country-specific standard deviation of these covariates.

6.2 Disaggregated analyses

In addition to the core metanalysis described here we will present the same analyses but conducted on all of the individual studies separately.

6.3 Controls

Versions of the core tests described in Table 3 but without the use of any covariates will also be reported.

6.4 Possible additional analysis of official data

For many studies official data on turnout and voting at the group level may become available. At this stage the granularity of this data is not known and, pending other official data, there is uncertainty about the polling station level dosage of interventions administered by the different studies. Official data has the advantage of being free of reporting biases (at least when elections are free and fair), but has the disadvantage of providing a noisy measure in cases with low dosage. The decision to include polling station areas for analysis using official data will be made as follows. Polling stations will be ordered, $1, 2, \dots, k, \dots, n$ in terms of treatment intensity (share of registered voters exposed to treatment T1) within each study (separately for the good news and bad news groups). Then, for each k the power to identify an effect as large as the estimated effect from the individual level analysis will be assessed, given an analysis including areas with density as large as k or greater. The largest group of polling station areas that collectively yield power of 50% or more will be included in this analysis. Note that with low dosages this set may be empty.
For any included sets the analysis will assess the effect of treatment as follows:
Define D_h as the share of cluster h (polling station area) individuals that *would* get treated if the unit were in treatment (dosage). Let $\overline{D}$ denote the (country specific) mean of D. Let $D' = D - \overline{D}$ denote D normalized to have a 0 mean. Then conditional on the polling station receiving good news (based on average values of $Q_i - P_{ij}$) estimate

$$y_h = \beta_0 + \beta_1 N_j^+ + \beta_2 T_h + \beta_3 T_h N_h^+ + \beta_4 T_h D_h' + \beta_5 D_h' + \sum_{j=1}^{k} (\nu_k Z_i^k + \psi_k Z_i^k T_i) + \epsilon_h \tag{6}$$

where y_h is the vote share for the incumbent, T_h is the treatment status of the cluster, N_j^+ is the cluster average of N_{ij}^+, normalized again to have 0 mean across clusters, the Z variables are cluster level controls, and ϵ_h is an error term. Here β_2 is the estimated treatment effect for a unit with average dosage. $\beta_1/\overline{D}$ is the estimated *individual level treatment effect* (under the assumption of no spillovers), generated from the polling station level data.
The analogous expression holds for bad news poling station areas.
In implementing this analysis we are conscious of the risk of ecological biases since the good news assessment is defined based on a group average but treatment effects may be drived by different individuals. As robustness check we plan to supplement this analysis with the same analysis but not conditioning on Q only and not P_{ij}. Good news areas for that analysis will be areas with performance equal to or above the median.

6.5 Bayesian hierarchical analysis model

A second analysis will employ Bayesian, multi-level meta-analysis techniques to allow for learning across cases and probe the sources of variation across cases. This approach requires stronger assumptions than the primary analysis but allows one to reassess the most likely estimates for each case in light of learning from other cases.
The simplest approach, drawing on a canonical model, is of the following form.
Say there are n_{1j} treated units and n_{0j} control units in study j. Let m_{1j} and m_{0j} denote the number of votes for the incumbent among treated and control units in study j respectively.
Then the data model is

$$m_{ij} \sim \text{Bin}(n_{ij}, p_{ij} \text{ for } i \in \{0, 1\} \tag{7}$$

This captures simply the idea that the number of votes in favor of the incumbent is a draw from a binomial distribution with a given number of voters and a given probability of supporting the incumbent in each arm of each study. Working on the logit scale we define parameters:

$$\beta_{1j} = \frac{1}{2}(\text{logit}(p_{1j}) + \text{logit}(p_{0j})) \quad (8)$$

$$\beta_{2j} = \text{logit}(p_{1j}) - \text{logit}(p_{0j}) \quad (9)$$

These correspond to the average support for the incumbent and the treatment effect of the informational intervention, respectively. We are interested especially in β_{2j} which corresponds to the average treatment effect in each study, on the log-odds scale.

Our priors on the collection of pairs (β_{1j}, β_{2j}) is given by a product of bivariate normal distributions with parameters α and Λ:

$$p(\beta|\alpha, \Lambda) = \prod_{j=1}^{7} N\left(\left(\begin{array}{c}\beta_{1j}\\ \beta_{2j}\end{array}\right)\middle|\left(\begin{array}{c}\alpha_1\\ \alpha_2\end{array}\right), \Lambda\right) \quad (10)$$

Here α_2 is of particular interest corresponding to the population analogue of β_{2j}.

For hyperpriors we assume uninformative uniform priors over $\alpha_1, \alpha_2, \Lambda_{11}, \Lambda_{22}$ and the correlation $\Lambda_{12}/(\Lambda_{11}\Lambda_{22})^{\frac{1}{2}}$.

The quantities we extract are the treatment effects for each study (with credibility intervals) as well as the posteriors on α_1, α_2.

In addition to this simple model we will report results from a second hierarchical logistic model that allows for systematic individual and study level variation in the same manner assumed in the core specification but allowing country level covariates to enter at the country level and cluster and individual level covariates enter at those levels. As with the core model, inverse propensity weights are included when non-uniform assignment propensities are employed. Again from this model study level average treatment effects will be estimated along with population parameters.

6.6 Exploratory analysis

In addition to the core tests described above, the analysis will engage in more exploratory analyses to assess how treatments altered the decisions voters took (using measure M7) as well as the comparability of effects across sites. For the latter analysis the country level treatment effects will be compared in light of the effects of treatment on mediators — that is, we will seek to report the shift in voting outcomes for units of treatment *scaled* in terms of the effects of treatment on mediators.

6.7 Learning about learning

One of the key tests of the usefulness of the *Metaketa* initiative is the extent to which the research and the policy communities learn from the aggregation of the coordinated studies. At the end of this *Metaketa*, we will gather a set of policymakers and academics, randomly divide them into samples, provide a briefing on the design of all studies, and then elicit prior beliefs about the effects of all studies. For treatment samples, we provide each with results from a random set of 5 of the studies, and incentivize them to provide updated expectations of results from the remaining studies. Some treatment samples will be encouraged (or required) to use predictive models while others will rely on subjective assessment and subject-matter knowledge. From this we expect to learn how results from some studies affect general beliefs, whether they make beliefs more accurate and how subjective inferences across studies fares relative to out-of-sample assessments of fitted models. The full analysis strategy for this component will be developed at a later stage.

7 Ethics

All projects in the Metaketa will abide by a common set of principles above and beyond minimal requirements (i.e. securing formal IRB approvals, avoiding conflicts of interest, and ensuring all interventions do not violate local laws):

- The egap principles on research transparency http://egap.org/resources/egap-statement-of-principles/
- Protect staff: Do not put research staff in harm's way.
- Informed consent: Subjects that are individually exposed to treatments will know that information they receive is provided as part of a research project. Core project data will be publicly available in primary languages at http://egap.org/research/Metaketa/
- Partnership with local civil society or governmental actors to ensure appropriateness of information
- Non-partisan interventions: Only non-partisan information will be provided where by non-partisan we mean that (1) it is coming from a non-partisan source; (2) it reveals information about performance of incumbents (candidates) regardless of their party.
- Approval from the relevant electoral commission when appropriate

The studies in general will not seek consent from individual politicians even though these may be affected by the interventions. The principle is that any information provided is information that exists in the political system that voters can choose to act upon or not and that this information is provided with consent, in a non-partisan way, without deception, and in cooperation with local groups, where appropriate.

8 Caveats

We are conscious of a number of limitations of this research design which will be relevant for interpretation of some results. Most important are:

1. Although we are in the good position of being able to assess comparable interventions in multiple sites, these sites are not themselves random draws from a population of sites. They reflect case level features such as the timing of elections and the feasibility of doing research as well as research team features such as researcher connections to these sites.
2. Although the information that is provided in different areas share many features they also differ in systematic ways (see discussion above).
3. Although there is reasonable statistical power in individual studies and in pooled analyses; power is weak for assessing some heterogeneous effects, especially those operating at the country level.
4. By design, with information provided to voters and treatment status not assigned at the politician level or made known to politicians, the effects estimated are partial equilibrium effects.
5. Although we gather data on the information available to voters prior to administration of treatment (in all studies with a baseline survey), we do not know what information voters receive between baseline and the vote. Thus estimates should be interpreted as intent-to-treat estimates even when treatment is delivered to all treatment units (and only those). Manipulation checks can be used to assess the extent to which treated and control units change beliefs between baseline and endline.

1.2 Study Designs

Table 1 summarizes key information on the individual study designs. Further designs details are available at `http://egap.org/metaketa/metaketa-information-and-accountability`, where links are provided to the pre-analysis plans for the individual studies.

Table S1. Individual study designs.

Country	Uganda 1	Uganda 2	Brazil	Mexico	Benin	Burkina Faso	India (as planned)
Location(s) within country	Central and Eastern Uganda	Countrywide	Pernambuco province	States of Guanajuato, México, San Luis Potosí, and Querétaro	Countrywide	Sahel, Centre-Nord, Plateau Central, Centre-Est, Centre-Sud, and Cascades regions	Bihar state
Governmental and non-governmental partners	Innovations for Poverty Action	Twaweza	Tribunal de Contas do Estado de Pernambuco (TCE-PE)	Qué Funciona para el Desarrollo (QFD); Borde Politico	Centre d'Étude et de Promotion de la Démocratie	Innovations for Poverty Action (IPA); Programme d'appui aux collectivités territoriales (PACT)	Sunai Consultancy
Intervention							
Information content	Candidates answering six questions on their policy positions and backgrounds, including: (a) their priority policy-area for the constituency; (b) their position on whether additional administrative districts should be created; (c) their position on the legal consequences for candidates convicted of vote buying; (d) their qualifications for running for office; (e) the personal characteristic they believe best prepares them for office; and (f) their past achievements	Information on local budget irregularities based on reports from the Office of the Auditor General: percentage of unaccounted-for funds in a citizen's district compared with unaccounted-for funds in other districts; also, examples of unaccounted-for funds when the district ranked below the national median on budget management, and examples of public projects that were managed well when district budgets had fewer irregularities than the national median	Information about whether municipal accounts were recently approved or rejected by TCE-PE auditors	Information on percentage of unauthorized/ misallocated spending (relative to municipalities governed by opposition parties in the same state) based on information from the Federal Auditor's Office (ASF)	Information on legislative performance of incumbent (relative to department and national averages), including: (a) rate of attendance at legislative sessions; (b) rate of posing questions during legislative sessions; (c) rate of attendance in committees; (d) productivity of committee work; information compiled into three indices	Information on municipal government performance (relative to other municipalities in the region) in areas of primary education, primary health care, water, sanitation, and administrative services	Information the criminal backgrounds of candidates for state assembly elections: number of criminal cases faced, and information on the average number of criminal cases faced by candidates in other constituencies in the same subdivision of the state
Mode of information delivery	Video displayed on tablet and presented to respondent by enumerator	SMS	Flyer during baseline survey, with enumerators providing oral summaries	Flyer	Video displayed on tablet and presented to respondent by enumerator in one of eight local languages	Flashcard presentation by enumerators, plus oral summaries	Flyers provided to respondents and explained (for 6 minutes) by survey enumerators; respondents asked to keep flyer visible in their household until the election
Timing of information delivery	1–2 weeks before election	One week before election	2–3 weeks before election	Between 3 weeks and four days before elections	Between 7 weeks and 2.5 weeks before election	1–3 weeks before election	One month before election
Alternative arm intervention	Public screenings of candidate videos in village meetings	Information on quality of local public goods and services (education, health, roads, water)	Information on municipality's performance in National Literacy Examination	Information publicly provided via loudspeakers, alongside common arm treatment	(a) Civics lesson on importance of legislative performance; (b) public provision of information in village meetings, with varying dosages	Personal invitation to attend a municipal council/special delegation meeting	Common arm information on politicians' criminal backgrounds delivered by influential local leaders rather than survey enumerators
Measurement	2	3	4	5	6	7	8
Definition of P (prior beliefs about substance of the information provided)	Respondents' prior beliefs about incumbents' policy positions and personal attributes, plus the importance (i.e. weight) they attach to each policy/attribute	Respondents' prior beliefs about budget management in their district relative to other districts (5-point scale: much worse, a little worse, no prior belief, a little better, much better)	Respondents' prior beliefs on whether their municipalities' accounts were accepted or rejected	None in primary analysis; but in robustness test, impute priors (measured at endline) from control precincts in same randomization block	Legislative performance index compared to department and nation (4-point scale)	How voters rank their own municipality relative to other municipalities in the region with respect to the quality of public services under the previously elected municipal government	N/A

Table 1 Continued from previous page

Country	Uganda 1	Uganda 2	Brazil	Mexico	Benin	Burkina Faso	India (as planned)
Detailed definition of Q (information provided to treatment group)	For policy (items a–c in information content row): extent of alignment between candidates' stated policy positions and respondents' prior beliefs about those positions<br>For attributes (items d–f in information content row): experts' average assessment of a candidate's performance in answering the questions	Budget management relative to other districts: much worse, a little worse, a little better, much better	Accounts accepted or rejected by TCE-PE	Percent misallocated spending, relative to municipalities in the same state governed by other parties (continuous measure)	Legislative performance index compared to department and nation (4-point scale)	How the municipality ranked relative to other municipalities in the region with respect to the quality of public services under the previously elected municipal government	N/A
Additional notes on P/Q and the coding of good/bad news	Per this study's pre-analysis plan, an overall P and Q is not calculated for each respondent; rather, Q-P is calculated for six subdimensions; these differences are averaged, weighting by the importance attributed to each dimension by respondents at baseline, yielding an overall measure of good and bad news; N.B. study PAP establishes coding rules for instances where P=Q	To compute Q-P, Q is converted to a 5-point scale, with the 3rd category always empty, to mirror the 5-point coding of P; thus if Q is a little worse and P is no prior belief, the information is bad news	Information coded as good news if accounts were accepted, and bad news otherwise; this adheres to the meta-preanalysis plan (MPAP) tie-breaking rule since the median value of Q in the sample is accepted	Information coded as good news when Q is above median, and bad news otherwise			Planned two ways of defining good/bad news. Bad news when: (a) incumbent faces more criminal charges than the mean/median candidate in the constituency; or (b) incumbent faces more criminal charges than the regional average; good news otherwise
Vote choice (M1) survey question	"As I said before, this is confidential. Which candidate did you vote for as the area MP, the member of parliament of your constituency?"	"If you voted for LC5 chairperson, what party does the candidate that you voted for represent?"<br>"If you voted for LC5 councilor, what party does the candidate that you voted for represent?"	"I would like you to take this ballot and secretly mark for whom you voted for mayor in those elections. When you finish, fold it and, without showing it, put the ballot in this ballot box."	"If you voted, for which party did you vote?"	"Now we would like to know more about the party you voted for in the legislative elections last Sunday. Your response is entirely confidential and will not be shared with anyone outside of our research team. We would like to know if you voted for the party of [PRINCIPAL DEPUTY NAME]. The name of that party is [PARTY NAME] and its symbol is [PARTY SYMBOL]. You need say only yes or no. Did you vote for the party of [PRINCIPAL DEPUTY NAME]?"	"As part of this survey, we now invite you to take part in a pre-election poll. This is not a real election, but a mock election. We do this so we can estimate how many votes the different parties are going to get in the actual municipal elections on May 22. We will make these results public, so that people can compare them with the official election results." I.e. respondents were asked to cast votes in a polling station simulation. For this purpose, mock polling stations were set up in the respondents' villages. The ballots were marked with identifier codes that were visible only under ultraviolet light. Through the invisible identifiers, the vote choices could eventually be linked to the anonymized survey data.	"Who did you vote for? I am giving you this slip, which has names and election symbols of all the candidates that you saw on the voting machine. On this slip please put a mark in front of the same symbol against which you pressed the button."
Turnout (M3) survey question	"While talking to people about (today's) parliamentary elections, we find that some people were able to vote, while others were not. How about you—were you able to vote or not?"	"Did you vote in the February 24, 2016 LC5 elections?"	"On October 2, there were elections for mayor, and as in any election, there are always people who were not able to vote. Did you vote in the elections of October 2?"	"On June 7 there were elections for the Municipal President and, as in any election, there are always people who do not have time to go to vote and others who do not care. Did you vote in the June 7 elections?"	"In the most recent legislative elections on April 26, some people were not able to vote. How about you—were you able to vote?"	"How likely is it that you will actually go to vote in the upcoming municipal elections?" (We recode answers as a dichotomous variable.)	"While talking to people about the recent elections to the Vidhan Sabha, we find that some people were not able to vote. How about you—were you able to vote or not?"

Table 1 Continued from previous page

Country	Uganda 1	Uganda 2	Brazil	Mexico	Benin	Burkina Faso	India (as planned)
Strategy for sampling constituencies and respondents (within constituencies)	Researchers selected 4 parliamentary constituencies (non-random); within each constituency, selected three polling stations with the greatest overlap between a polling station catchment area and its main village (non-random); at least 85 registered voters selected in each polling station/village (random)	Implementing partner selected 27 districts (random within region blocks); 762 villages selected with these districts (random); 31,310 individuals recruited into sample via collective gatherings and door-to-door visits (non-random)	All 7 municipalities where accounts were rejected and incumbents ran for re-election (non-random); for municipalities where accounts were accepted, eliminated the smallest municipalities and the state capital (non-random), grouped remaining 75 municipalities into two strata based on information conveyed in alternative arm, and sampled an equal number of municipalities from each stratum (random, with inclusion probabilities proportional to 2010 census population)<br>For respondents, sampled census tracts with probability proportional to the number of households in the 2010 census (random), excluding least populous and most rural tracts (non-random); in municipalities where account was approved, sampled 5 census tracts and 8 households per tract (random); in municipalities where accounts were rejected, sampled between 5 and 26 census tracts, 16 households per tract (random)	Researchers selected 4 low-violence states displaying internal variation in municipal electoral performance of different parties, as well as variation in performance on ASF audits (non-random); within these states, selected 26 low-violence municipalities where an audit was released in 2015, and for which malfeasance measures varied across parties. Ensured that sample matched the distribution of incumbent parties across audited municipal governments (non-random). Within each municipality, sampled up to one third of precincts, oversampling rural and sparsely populated areas; selected 10 voters from each treated precinct and 100 from each control precinct (random)	Researchers selected 30 communes for which a one-to-one matching of incumbent to commune could be identified, and where incumbent was running for re-election (non-random); 225 villages selected within these communes (random); 20 respondents selected in each village (random)	Researchers selected 39 municipalities that were part of a previous study and where the incumbent party was re-elected in 2012 (non-random); 146 villages sampled from these municipalities (random); selected up to 12 individuals per village (random)	Researchers selected 25 secure assembly constituencies where both competitive candidates were facing serious criminal charges in 2010 (non-random); selected 24 polling booths per constituency (random); planned to select 20 voters per polling booth (random)
Timing of baseline survey	1–2 weeks before election	One month before election	2–3 weeks before election	No baseline administered	Conducted concurrently with intervention, between 7 weeks and 2.5 weeks before election	Immediately prior to the information treatment	Conducted concurrently with intervention, one month before election
Timing and mode of endline survey	Phone survey; started on election day (February 18, 2016), ended two weeks later.	Phone survey; started a day following the February 24, 2016 elections, ended after 5 days.	In-person survey; started 17 days after election, ended one month after election.	In-person survey; started 5 days after the June 7, 2015 election, ended after 25 days.	Phone survey; started a day following the April 26, 2015 election, ended after 3 weeks.	Immediately after the information treatment (post-treatment survey and polling station simulation)	Not conducted
Endline attrition rate (relative to baseline)	11 percent	22 percent	19 percent	N/A, since no baseline administered	44 percent	5.6 percent	N/A
Randomization	2	3	4	5	6	7	8
Unit of randomization for common arm	Individual	Individual	Individual	Electoral precinct	Village/urban quarter	Individual	Polling booth
Randomization blocks for common arm	Constituency	Village	Census tract	Within urban/rural, block according to 23 social, economic, demographic, and political variables	3 blocks defined within each commune: (a) urban electorally competitive; (b) rural, electorally competitive; (c) rural, not electorally competitive	Village	Assembly constituency
Equal probability of assignment within blocks?	Yes	No; inverse propensity weights employed	No; inverse propensity weights employed	Yes	Yes	Yes	Yes
Ethics	2	3	4	5	6	7	8

Table 1 Continued from previous page

Country	Uganda 1	Uganda 2	Brazil	Mexico	Benin	Burkina Faso	India (as planned)
IRB approval	Yale University Protocol 1504015711 Stanford University Protocol 33547 Uganda National Council for Science and Technology Protocol SS3781 Mildmay Uganda Research and Ethics Committee Protocol 0104-2015	UCSB Protocol 15-0690	MIT Protocol 1604551604; Boston University Protocol 4094X; Universidade Federal de Pernambuco Centro de Ciencias da Saude/UFPE Protocol 1.571.592	New York University Protocol 15-10587; Harvard University Protocol IRB15-1008	UCSD Protocol 141034S; Harvard Protocol IRB14-3188; George Washington University Protocol 90140; Texas A&M University Protocol IRB2014-0643	Yale University Protocol 1405013896; IPA Protocol 14159	Dartmouth College Protocol STUDY00028849
Government permissions	Consent of political parties and the Electoral Commission	Office of the President	Partnered with TCE-PE, the government agency conducted municipal audits	States' electoral institutes were informed	Obtained permission from the SG of the National Assembly	Ministry of Territorial Administration and Decentralization (MATD); Independent National Electoral Commission (CENI)	Election Commission of India (approval subsequently withdrawn)

1.3 Examples of Information Delivered

The MPAP (reproduced in section 1.1) briefly describes the type of information delivered in each study's common intervention arm (see the MPAP's section 2.1). One aspect is incorrect: while the MPAP states that scorecards were used to deliver information in the Uganda 1 study, in fact information was delivered through videotaped "Meet the Candidate" screenings that featured candidates for office, including incumbents. The distinction between the primary and alternate intervention arm is thus whether these videos were watched privately by individual citizens (common arm) or in public settings in villages (alternate arm). (Platas and Raffler (2018) also compare effects in primary and general elections, but the common intervention arm includes only screenings for primary elections.)

This section offers examples or illustrations of the information content delivered in each study (except India, which was not completed). For more extensive discussion of the interventions, see also the pre-analysis plans for the individual studies available at
`http://egap.org/metaketa/metaketa-information-and-accountability`.

Benin

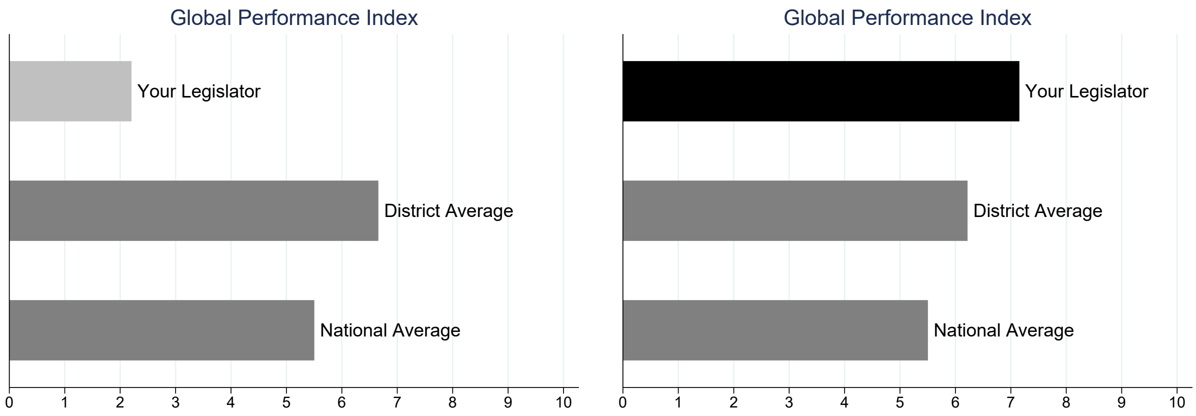

Fig. S1. Benin—Graphical representation of provided information.

Brazil

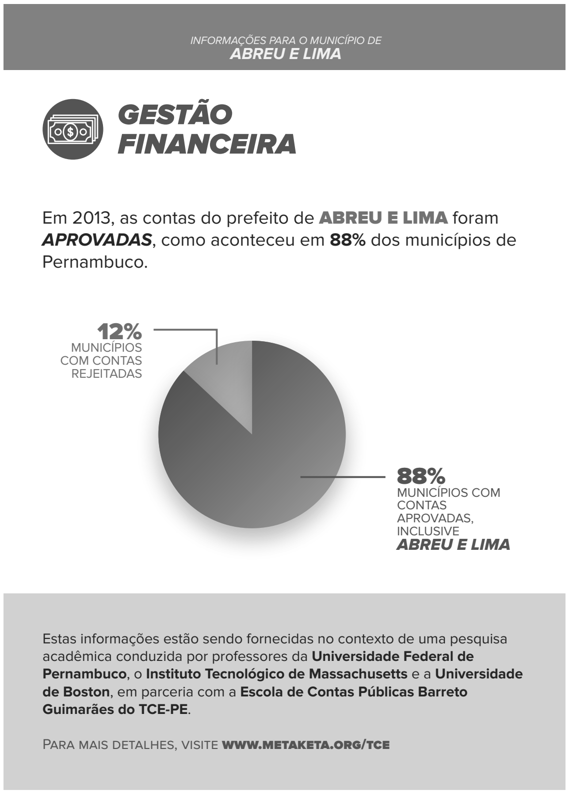

(a) Accounts Approved Flyer

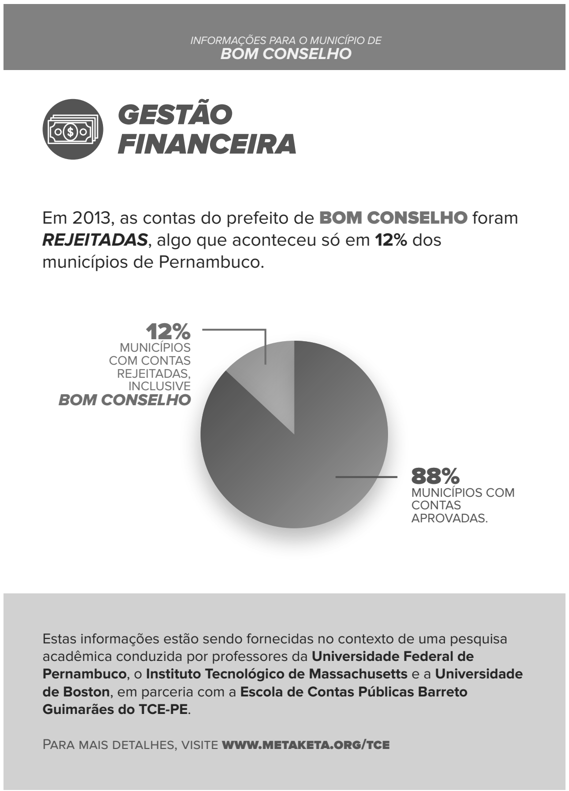

(b) Accounts Rejected Flyer

Fig. S2. Brazil—Flyers distributed to voters.

Burkina Faso

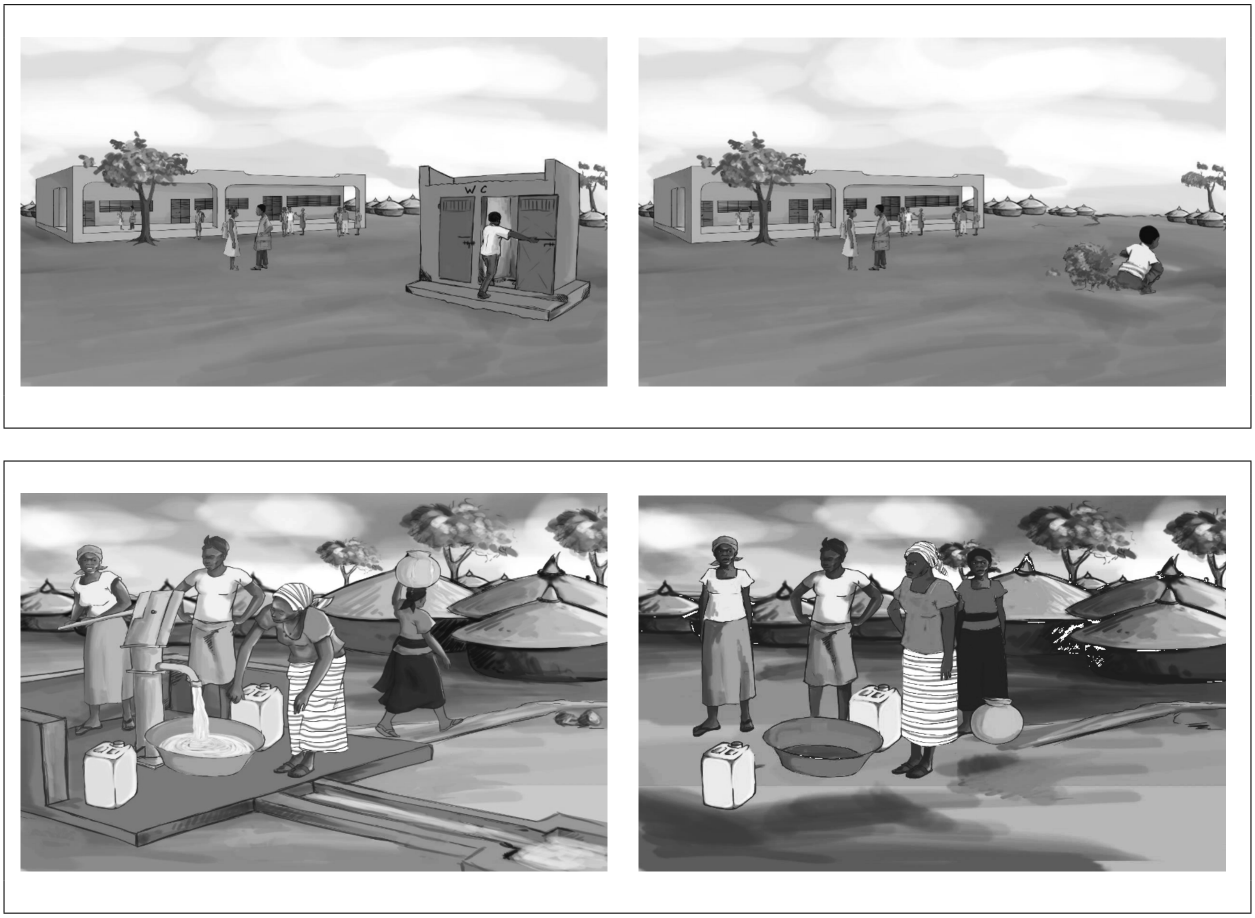

Fig. S3. Burkina Faso—Flashcard illustrations of municipal performance indicators. Top panel: Provision of school latrines. Bottom panel: Provision/maintenance of water points.

Mexico

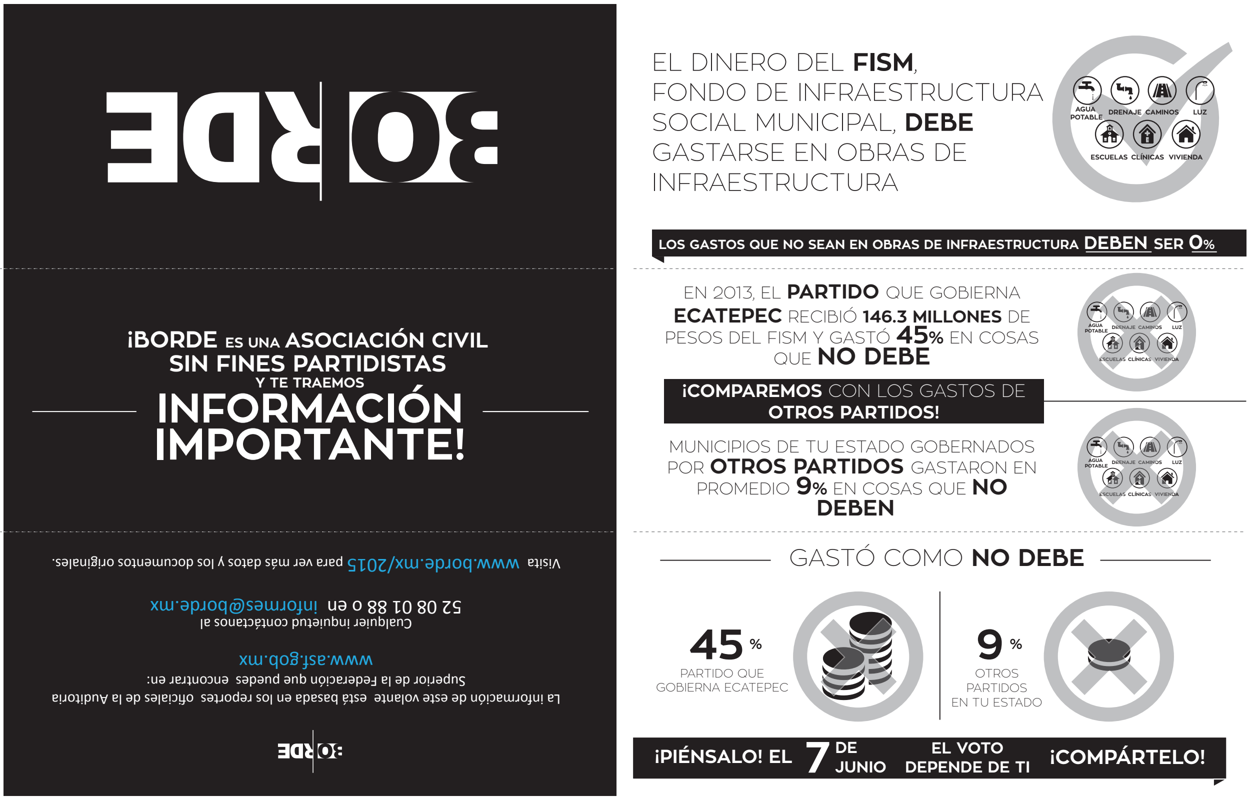

Fig. S4. Mexico—Example of benchmarked leaflet in Ecatepec de Morelos, México.

Uganda 1

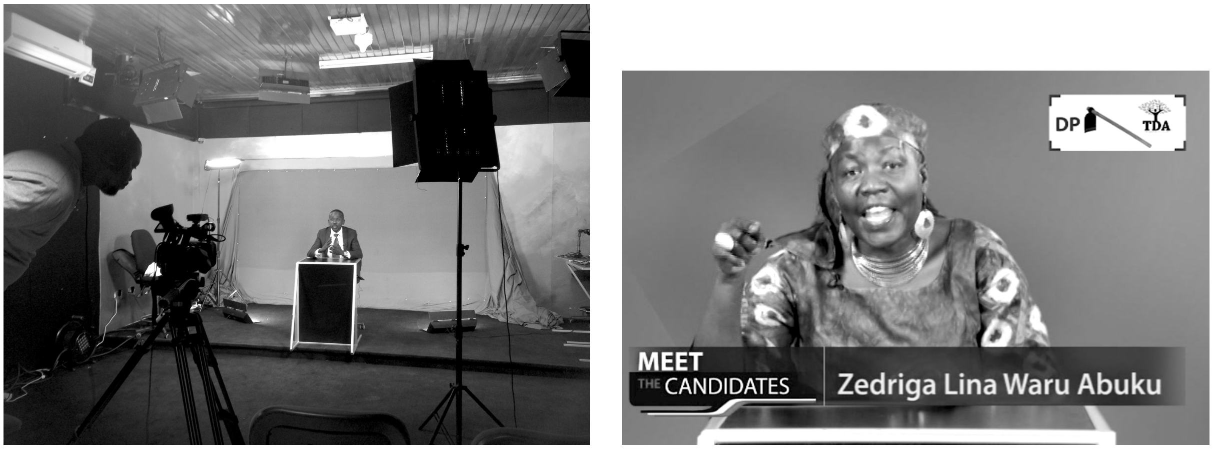

Fig. S5. Uganda 1—Candidate answering questions during a recording session and candidate as seen in video.

Uganda 2

The following are examples of text messages sent via mobile phones to voters:

- "The Auditor General conducts yearly audits to record instances where LC Vs could not satisfactorily explain how its money has been spent."
- "Unexplained spending is often an indicator of mismanagement, fraud or poor quality services."
- "Your LC V did much worse than most other LC Vs in the recent audit."
- "In your LC V, the auditor found issues with 120 million UGX from its budget of 19 billion UGX. This is much worse than in other districts."
- "This means that 6.3 out of 1000 UGX in your LC V budget had issues. In most LC Vs, 2.2 out of 1000 UGX had issues. Your LC V did much worse than average."
- "One reason your LC V did much worse than average is that payments of 98 million UGX were made without proper documentation."
- "Another reason your LC V did much worse than average is that a bid for borehole construction included unexplained expenditures."

1.4 Descriptive Statistics

Table S2. Descriptive statistics for sample of good news.

Statistic	N	Mean	St. Dev.	Min	Max
Nij	19,400	1.650	1.156	0.000	4.000
Voter turnout	16,037	0.801	0.399	0	1
Effort	13,237	2.396	0.944	1	4
Dishonesty	13,756	2.458	1.209	1	5
Backlash	2,157	0.232	0.309	0.000	1.000
Age	20,020	35.461	12.729	17	99
Co-ethnicity	17,382	0.665	0.472	0.000	1.000
Education	20,033	7.191	4.108	0.000	20.000
Wealth	19,903	2.772	1.091	−2.317	5.000
Co-Partisanship	16,550	1.155	1.797	0	9
Voted in past election	20,015	0.827	0.378	0.000	1.000
Secret ballot	19,788	2.098	1.420	1.000	5.000
Free and fair elections	19,144	3.344	1.506	1.000	5.000

Note: * $p < 0.05$; ** $p < 0.01$; *** $p < 0.001$.

Table S3. Descriptive statistics for sample of bad news.

Statistic	N	Mean	St. Dev.	Min	Max
Nij	19,191	−1.077	1.031	−4.000	0.000
Voter turnout	15,597	0.798	0.401	0	1
Effort	12,761	2.597	0.938	1	4
Dishonesty	13,589	2.481	1.239	1	5
Backlash	2,339	0.147	0.192	0.000	1.000
Age	19,584	37.370	13.345	18	92
Co-ethnicity	16,749	0.815	0.388	0.000	1.000
Education	19,604	6.657	3.968	0.000	20.000
Wealth	19,260	2.890	1.057	−2.805	5.000
Co-Partisanship	17,002	1.151	1.675	0	9
Voted in past election	19,568	0.862	0.345	0.000	1.000
Secret ballot	19,281	2.401	1.407	1.000	5.000
Free and fair elections	18,966	3.567	1.442	1.000	5.000

Note: * $p < 0.05$; ** $p < 0.01$; *** $p < 0.001$.

1.5 Balance Tests

Table S4. Balance of covariates.

Baseline covariate	Control mean	Treat mean	d-stat	$\hat{\beta_1}$	$\hat{\beta_2}$	N
Prior	1.35	1.38	0.03	0.05	0.02*	20617
	(1.26)	(1.28)		(0.01)	(0.01)	
Good news	0.48	0.48	0	-0.1***	-0.01***	23803
	(0.5)	(0.5)		(0.02)	(0.01)	
Gender	0.43	0.42	-0.02	0.01*	-0.02***	23998
	(0.5)	(0.49)		(0.01)	(0.01)	
Age	39.56	39.62	0	0***	0***	23917
	(14.96)	(14.96)		(0)	(0)	
Co-ethnicity	0.65	0.63	-0.03	0***	-0.03***	19391
	(0.48)	(0.48)		(0.01)	(0.01)	
Education	5.45	5.43	0	0***	0***	23960
	(4.79)	(4.71)		(0)	(0)	
Wealth	2.42	2.41	-0.01	0.02*	0.01*	23693
	(1.44)	(1.42)		(0.01)	(0)	
Co-Partisanship	3.64	3.6	-0.01	0.06	0***	20025
	(2.81)	(2.78)		(0)	(0)	
Voted in past election	0.78	0.77	-0.01	0.07	0.17	23892
	(0.42)	(0.42)		(0.01)	(0.01)	
Voted incumbent past election	0.66	0.66	0	0.22	0.03*	19869
	(0.47)	(0.47)		(0.01)	(0.01)	
Clientelism	1.99	1.96	-0.02	-0.04***	0***	22911
	(1.41)	(1.41)		(0)	(0)	
Salience of information	0.52	0.54	0.03	-0.04***	0***	20143
	(0.5)	(0.5)		(0.01)	(0.01)	
Credibility of information	0.41	0.43	0.05	-0.02***	-0.01***	21415
	(0.49)	(0.5)		(0.01)	(0.01)	
$\Pr(\chi^2)$	0.1					

Note: Results show the control and treatment means for each of the pre-treatment covariates. Means and standard deviations are weighted by block share of non-missing observations. *d*-stat is calculated as the difference between treatment and control means normalized by one standard deviation of the control mean. $\hat{\beta_1}$ ($\hat{\beta_2}$) is the coefficient in a regression of vote choice (turnout) on each covariate separately, in the control sample. As with main specification, we include randomization block fixed effects and standard errors clustered at the level of treatment assignment. We also show the probability of rejecting the null that none of the covariates is predictive of treatment. All regressions include block fixed effects, standard errors clustered at the level of assignment and inverse propensity weights, and all countries are weighted equally. * $p < 0.05$; ** $p < 0.01$; *** $p < 0.001$.

1.6 Power Analysis

Calculating the power of our meta-analysis is somewhat difficult since there are many blocks and clusters of unequal size, complex assignment schemes—different in different studies—and complex estimation involving inverse propensity score weights, country weights, and clustered standard errors; moreover, the average effects of interest are averages over heterogeneous effects that depend upon our specification of good news and bad news groups. Off-the-shelf power calculators are not able to deliver estimates of power for designs like this.

Nevertheless, power calculations are possible using an ex-post simulation approach, at least conditional on a model of the data-generating process. We implement this approach using the `DeclareDesign` package, in which we formally declare our data structure, our conjectured data generating process, our assignment schemes, our estimands, and our estimation strategy. We then use Monte Carlo simulations to "run" the design many times and assess statistical power—that is, the fraction of runs in which we reject a false null hypothesis—conditional on different conjectures about the size of the true effect. We note that a bonus of this approach is that we can check that our estimates are unbiased, given our design. This is a nontrivial question since the estimation strategy had to be tailored to match different assignment strategies used in different sites. Moreover, unbiasedness is not guaranteed given heterogeneous sized clusters in some studies. The results from the "diagnosis" of this design suggest no bias concerns.

The most important feature of the power analysis involves the specification of a data-generating process. We assume that an individual in block b and cluster c will vote for the incumbent with probability p^0_{bc}, where p^0_{bc} is drawn from a distribution centered on the observed *block level* share supporting the incumbent in the control group, with a variance that produces an intra-cluster correlation coefficient (conditional on block, b) approximately equal to the observed correlation in that group.

For any stipulated effect δ we assume that individuals support the incumbent in the treatment condition with probability p^1_{bc}

$$p^1_{bc} = \phi\left(\phi^{-1}(p^0_{bc}) + \delta N_i\right) \tag{1}$$

where ϕ is the standard normal density and ϕ^{-1} its inverse. The approach here then assumes that treatment induces a constant effect (conditional on the value of N_i) on a latent support variable that determines the propensity to support the incumbent. For instance, for $\delta = 1$ an individual that supports an incumbent with probability $p^0 = .5$ in control and for whom $N_i = 1$, would support them with probability 0.84 in treatment (i.e. $\phi(0 + 1)$). In practice, a probit-type approach is employed, in which an individual has a normally distributed shock e_i and votes for the incumbent if e_i falls below $\phi^{-1}(p^t)$ for condition t; this ensures that in realizations individuals with positive effects have non-negative changes in their votes. Note that for any specified δ, different individuals have heterogeneous effects that depend upon the propensity in their control condition and their own value of N_i. Given all these different propensities across all individuals, the estimand of interest is the average difference in voting propensity, across studies, for individuals in treatment and control. To calculate power, we consider a range of possible δs and for each one calculate the implied estimand and the probability that our estimate of that estimand will be statistically significant. Results are presented in Figure 6.

We see that power for different average effects depends on the outcomes of interest. For the electoral support outcomes, we hit 80 percent power for average treatment effects of around 5 percentage points; for the turnout quantities, we would hit power of 80 percent with effects of around 4 percentage points. In other words, to register a statistically significant result on our primary outcome in 80 percent of repeated hypothetical experiments, the interventions would have to change the vote choice of 5 out of every 100 voters. Together with the tightness of our observed confidence intervals, we see these results as evidence that null results were not forgone conclusions.

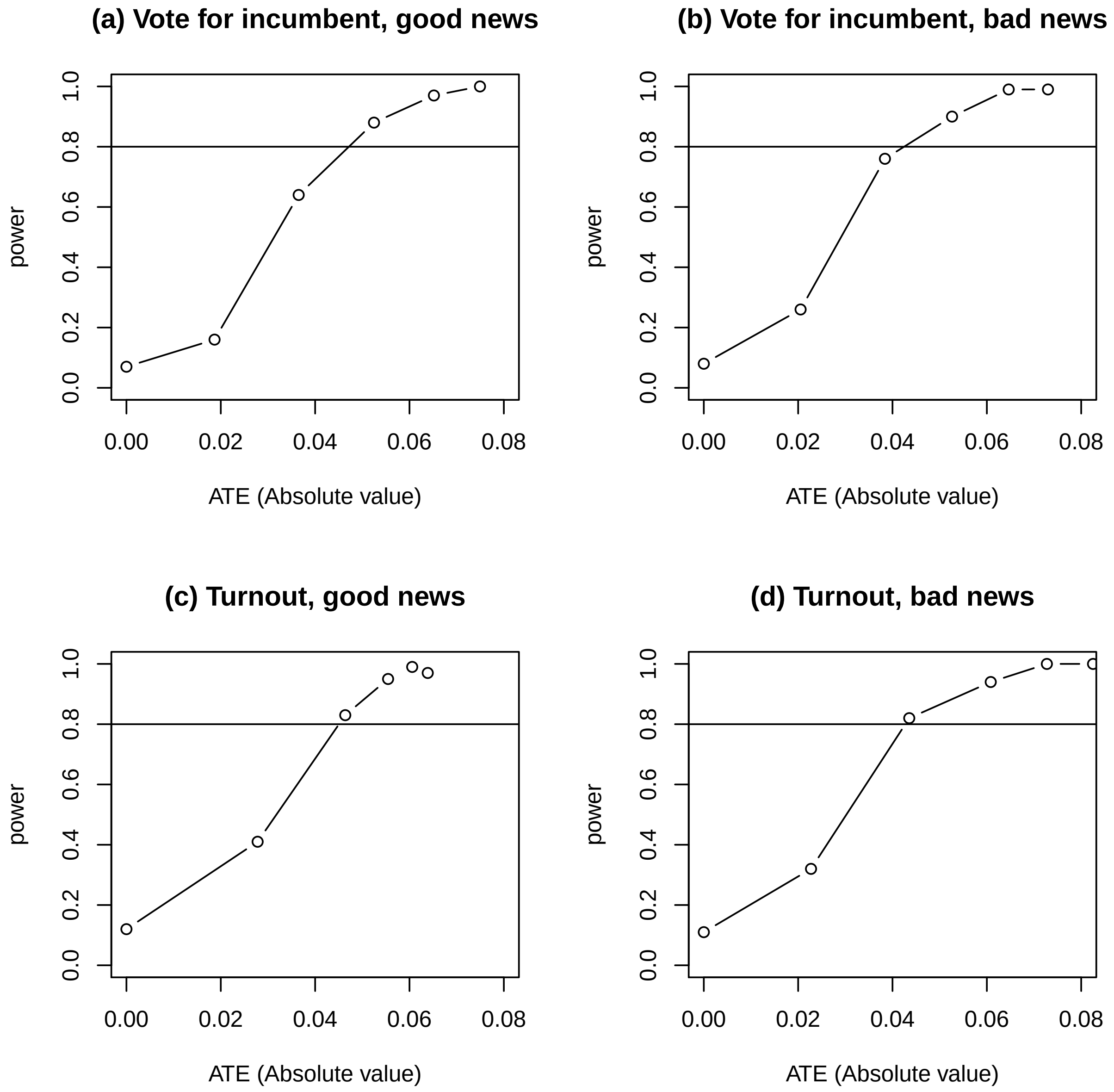

Fig. S6. Power analysis of minimal detectable effects, computed using Monte Carlo simulation. The horizontal axis varies the conjectured average treatment effect, while the vertical axis shows statistical power: the probability of rejecting the null hypothesis at $\alpha = 0.05$.

Section S2. Primary analysis: Robustness and reliability of results

2.1 Additional Test on Average Effects Across Cases

Table S5. Effect of information, conditional on distance between information and priors, on vote choice, and turnout.

	Vote Choice		Turnout		Vote Choice	Turnout
	Good News	Bad News	Good News	Bad News	Overall	
	(1)	(2)	(3)	(4)	(5)	(6)
Treatment	0.0002	−0.003	0.002	0.018	0.003	0.017*
	(0.015)	(0.015)	(0.013)	(0.012)	(0.010)	(0.008)
N_{ij}	−0.015	−0.049***	0.002	0.011	−0.050***	0.010
	(0.016)	(0.015)	(0.014)	(0.013)	(0.012)	(0.011)
Treatment * N_{ij}	−0.012	−0.001	−0.002	0.0001	−0.002	−0.002
	(0.020)	(0.020)	(0.019)	(0.015)	(0.012)	(0.011)
Control mean	0.355	0.398	0.843	0.835	0.368	0.837
RI p-value	0.994	0.848	0.89	0.18	0.81	0.057
Joint RI p-value	0.972		0.309			
Covariates	Yes	Yes	Yes	Yes	Yes	Yes
Observations	13,190	12,531	14,494	13,148	25,814	27,731
R^2	0.298	0.281	0.200	0.160	0.274	0.165

Note: "Vote choice" indicates support for the incumbent candidate or party. Standard errors are clustered at the level of treatment assignment. Pooled results exclude non-contested seats and include vote choice for LCV councilors as well as chairs in the Uganda 2 study (see Table 6, below, for further explanation). This means each respondent in the Uganda 2 study enters twice, and we cluster the standard errors at the individual level. We include randomization block fixed effects and a full set of covariate-treatment interactions. Control mean is the weighted and unadjusted average in the control group. * $p < 0.05$; ** $p < 0.01$; *** $p < 0.001$

2.2 Deviations from Pre-Registered Analyses

Our meta-pre-analysis plan (MPAP) was unclear or incomplete with respect to several data-analytic decisions, allowing for ex-post discretion; in two cases, it was methodologically incorrect, suggesting desirable deviations from the pre-specified analysis. Different possible ways of handling study-specific analytic choices also introduced ex-post discretion.

Table 6 documents the registered choices (or the area for which the registered MPAP was unclear, incomplete, or incorrect); each deviation or ex-post analytic choice; and the rationale for each deviation or ex-post decision. We assess the sensitivity of overall results to these choices in section 2.3.

Table S6. Deviations from MPAP and study PAPs in the meta-analysis.

**Registered Analysis in MPAP**	**Deviation**	**Rationale**
Cluster standard errors on politician j	Cluster standard errors on the unit of treatment assignment	Registered analysis incorrect, since the target parameter is the effect for our study group of politicians (politicians are not a random sample from a larger population)
No specification of hypothesis testing by randomization inference (RI)	Report RI p-values in all tables and treat as primary tests	Ambiguity in MPAP; preference for design-based tests
No specification of study-level weighting	Results with equal study-level weighting (primary) as well as unweighted analysis (secondary)	Average study-level effect is important estimand; without weighting, studies with larger samples contribute more to estimate
For six hypothesis families, present joint RI p-values (see text) and tests employing false discovery rate (FDR) correction, in addition to nominal p-values	Present joint RI p-values and nominal p-values, but not FDR correction	For primary meta-analysis, all estimated effects insignificant at conventional levels
Pre-specified hypotheses about intermediate outcomes and moderators, employing data from all studies; 14 baseline covariates to increase statistical precision	Most secondary hypothesis tests conducted on incomplete data; only 10 pre-treatment covariates used for adjustment	Not all pre-specified variables were gathered by all teams
Analysis that assesses the distribution of effects based on the count of votes for the incumbent and the total number of voters in each study	Employ approach proposed in Rubin (1981), using study level estimates and standard deviations as inputs	MPAP specification at odds with the design, since it does not take account of the fact that the treatment was randomized within blocks; accounting for this would require a more complex multilevel structure with block and country effects; we use a closely related model that is simpler in structure but similar in spirit
**Mexico PAP**		
Procedure for estimating block-level priors using the control group	Primary meta-analysis ignores priors (Q only), although robustness checks use difference in individual-level posteriors and block-level priors	Baseline data could not be collected due to budget constraints; block-level priors measured on different scale from Q
**Uganda 1 PAP**		
Definition of good news/bad news based on aggregating across six subdimensions; $N \neq 0$ when $P = Q$ within subdimension	Uganda 1 PAP's good news/bad news coding retained for primary meta-analysis, but robustness checks use a good/bad news coding where $N = 0$ when $P = Q$ within subdimension	MPAP unclear on how to handle definition of N for Uganda 1; study PAP is clear
**Uganda 2 PAP**		
No restrictions on study group: analysis of all sampled respondents	Restriction to contested constituencies (but sensitivity to their inclusion examined via specification curve in section 2.3 and online Shiny App)	Electoral contestation is arguably a necessary condition for political accountability
No stipulation of politician type for common-arm analysis in MPAP or study PAP	Inclusion of LCV chairs and councilors in primary analysis, clustering standard errors on respondent, and including a fixed effect for councilors; chairs- and councilors-only analysis examined in specification curve (section 2.3) and online Shiny App	No consensus on what was intended thus err on side of inclusion
No stipulation of common arm treatment	Budget treatment (rather than public services treatment) considered as common intervention arm	Recollection of intent by Uganda 2 team and Metaketa I executive committee
No stipulation of level of office for common arm analysis	Meta-analysis focuses on LCV and ignores LCIII	Recollection of intent by Uganda 2 team
Unequal treatment assignment propensities inherent in multi-stage randomization not discussed	Meta-analysis implements inverse probability weights	Unweighted estimator can yield a biased estimate of the average treatment effect
**Burkina Faso PAP**		
Vote choice outcomes defined for those who did not intend to turn out to vote	Vote for incumbent (M1) recoded as 0 if turnout = 0	Follows M1 coding in MPAP

2.3 Specification Curve Analysis

How sensitive are our findings to the deviations and discrepancies described in Table 6? To answer this question as comprehensively and succinctly as possible, we implement the specification curve analysis reported in the text. Thus, we first identified the set of decisions having to do with dataset construction and modeling that we took in the course of performing the meta-analysis, including centrally those in Table 6 as well as areas in which the MPAP proposed more than one strategy (for instance, inclusion or exclusion of covariates). We also include in our specification curve an unregistered "leave one out" analysis in which we calculate the overall meta-analysis estimate, excluding one study at a time. From this we identify the exhaustive set of possible specifications; for every possible specification, we estimate a statistical model. In online materials, we present a flexible interface (an *R*-based Shiny App) that allows users to vary these specification choices themselves and assess the sensitivity of results; see www.egap.org/content/metaketa-i-interactive-meta-analysis.

In the specification curve analysis, we plot estimates from the full set of models for our primary outcome, vote choice (Figure 4 in the text) and our secondary outcome, turnout (Figure 5 in the text), with the plot in the top panel showing estimated effects of good news and the bottom panel showing bad news. For each plot, the horizontal axis depicts the estimated average treatment effect. The vertical axis lists the set of decisions. Decisions all come in pairs (e.g. unadjusted vs. covariate-adjusted analysis), with the exception of the leave-one-out analyses, which involves a set of seven options. Within the row associated with a particular decision, that decision is held fixed, and estimates from all other possible specifications—i.e., specifications based on all combinations of other decisions—are then presented. Thus each vertical dash in the body of the plot denotes a point estimate for a single model. We darken those estimates that are nominally statistically significant at the 0.05 level. The first row of each plot shows the collection of estimates from all specifications and thus depicts the overall proportion of estimates that are nominally significant.

The results are telling. For one of the plots—good news/turnout (Figure 5, Panel A in the text) we do not estimate a single statistically significant effect in the meta-analysis, underscoring the robustness of our overall null results in this case. For good news/vote choice (Figure 4, Panel A in the text), significant effects do materialize in a small set of specifications (55 out of 18,886), yet only when we weight all studies equally, when we do not not adjust for pre-specified covariates, and a majority of the time (98.2% of cases) when a particular study (Benin) is omitted.

For bad news vote choice (Figure 4, Panel B in the text), the treatment effect estimate is significant in 0.6 percent of specifications. These all occur in specifications which exclude the Burkina Faso study. They also all occur when we make certain specification choices related to the Uganda 2 study: excluding candidates who switched parties, including LCV councilors. In addition, most significant estimates occur when we restrict the analysis to competitive races in the Uganda 2 study (88.9% of significant estimates include the latter choice). The results for bad news/turnout depicted in Figure 5, Panel B in the text show the most evidence of impact, though even then in a minority (10.3 percent) of specifications. Here, we observe significant effects across a greater range of specifications. Unlike the bad news vote choice case, none of these choices is decidedly needed to obtain any significant estimates.

While we emphasize that the effect in our primary specification remains statistically insignificant, the specification curve provides suggestive evidence that disseminating bad news to voters about a sitting politician may spur them to turn out to vote. In another unregistered analysis, we also see hints that non-voters exposed to bad news may turn out to vote against the incumbent, although we cannot confidently reject the null of no effect. For this "vote against" analysis, the dependent variable equals 1 if a citizen votes for the opposition and 0 if she votes for the incumbent or does not turn out. (Our pre-registered outcome equals 1 if a citizen votes for the incumbent and 0 if she votes for the opposition or does not turn out to vote; see the MPAP's section 4.1). In sum, the results suggest the robustness of the null results in our meta-analysis.

2.4 Uncompleted India Study

Could study-level attrition account for our null overall results? One virtue of our pre-specification of studies and of integrated publication is that they make implementation failures—and missing studies—evident. This is an advantage from the point of view of transparency, as it counters an under-recognized file drawer problem in experimental research. Missing studies limit our ability to draw inferences to the whole study group. Our planned India study did not occur due to local political backlash. If politicians correctly anticipated large effects of our informational interventions in that context—and in consequence moved to block implementation of the study—this could indicate that treatment effects would have been larger in India, had the study occurred.

To evaluate this question, we conduct a sensitivity analysis. (We are grateful to Fredrik Sävje for his advice on the approach we use in this subsection). We ask the following question: how big (in absolute value) would the estimated effect in India need to have been to produce a non-null estimated effect in the overall meta-analysis, given the findings of our other studies?

We can answer this question with some algebra. Let $\widehat{\mu}$ be the average estimated effect in the six realized studies, $\widehat{\theta}$ be the estimated effect in India had the study taken place, and $\widehat{\gamma}$ be the average effect we would have estimated had all seven studies taken place. Thus

$$\widehat{\gamma} = (6\widehat{\mu} + \widehat{\theta})/7 \tag{2}$$

and its estimated standard error is

$$\widehat{\sigma_{\widehat{\gamma}}} = \sqrt{36\widehat{\sigma_{\widehat{\mu}}}^2 + \widehat{\sigma_{\widehat{\theta}}}^2}/7 \tag{3}$$

where $\widehat{\sigma_{\widehat{\mu}}}^2$ is the estimated variance of $\widehat{\mu}$ and $\widehat{\sigma_{\widehat{\theta}}}^2$ is the estimated variance of $\widehat{\theta}$. (This is because $\text{Var}(\widehat{\gamma}) = \text{Var}[\frac{6\widehat{\mu}+\widehat{\theta}}{7}] = \frac{36\text{Var}(\widehat{\mu})+\text{Var}(\widehat{\theta})}{49}$; we replace $\text{Var}(\widehat{\gamma})$ with the estimate $\widehat{\text{Var}}(\widehat{\gamma})$, and the square root is the standard error. This calculation assumes independence of the effect estimates across countries. We took many measures to ensure that results in one study would not affect others—for example, by blinding researchers to results in other studies until all studies had been completed). The t-statistic is given by $\widehat{\gamma}/\widehat{\sigma_{\widehat{\gamma}}} = 6\widehat{\mu} + \widehat{\theta}/\sqrt{36\widehat{\sigma_{\widehat{\mu}}}^2 + \widehat{\sigma_{\widehat{\theta}}}^2}$. Thus, the t-statistic for the estimated average treatment effect across the seven studies would have been greater than 1.96 if and only if the estimated effect in India had satisfied the following inequality

$$\widehat{\theta} \geq 1.96\sqrt{36\widehat{\sigma_{\widehat{\mu}}}^2 + \widehat{\sigma_{\widehat{\theta}}}^2} - 6\widehat{\mu} \tag{4}$$

These calculations allow us to place bounds on how large the estimated treatment effect in India would have needed to be to produce a statistically significant result in the meta-analysis. First, assume an SE of 0.012 in India (that is, 1.2 percentage points for the 0-1 vote choice variable); this is the smallest of the study-specific standard errors seen in our baseline specifications. (Note that this assumption is likely to be conservative, since the India study clustered treatment assignment at the polling station level. Considering only the common intervention arm and the control group, there were to be 400 polling stations with 20 citizen respondents in each polling station; see Chapter 10 and the India team's PAP). This implies that in the good news case with our primary outcome of vote choice, we would have needed an estimated average treatment effect of 0.172, or 17.2 percentage points, to see a significant effect in the seven-study meta-analysis. We can perform the same calculation inputting the largest country-specific standard error (0.065). Under this assumption, we would have needed an estimated average treatment effect of 0.212—that is, 21.2 percentage points—for the seven-study meta-analysis to register a finding statistically distinguishable from zero. (In this case, $\widehat{\mu} = 0.001$ and $\widehat{\sigma_{\widehat{\mu}}}^2 = (0.015)^2$). These are enormous effects–an order of magnitude bigger than anything we see in other studies, including those, like Mexico, where we also see evidence of politician backlash to the treatment implementation. Even in the case where we see the largest $\widehat{\mu}$—in the bad news case with our secondary outcome, turnout—we calculate that we would have needed an estimated treatment effect in India of between 4.1 and 7.1 percentage points to see a significant effect in the overall estimate.

2.5 Tests of Differential Attrition

Table S7. Differential attrition.

	Vote Choice			Voter Turnout		
	Estimate	Std. Error	p-value	Estimate	Std. Error	p-value
Treatment	0.00	(0)	0.57	0.00	(0)	0.71
F-stat		13.78			15.26	
Pr(F)		0.39			0.29	

Note: Table shows the effect size of treatment on data missingness in incumbent vote choice and voter turnout across the entire sample. Pr(F) shows the probability of rejecting the null that none of the covariates is differentally determining attrition across treatment and control conditions. All regressions include block fixed effects, standard errors clustered at the level of assingment and inverse propensity weights, and all countries are weighted equally.* $p < 0.05$; ** $p < 0.01$; *** $p < 0.001$.

Section S3. Secondary analysis: A Bayesian approach

An alternative approach to meta-analysis takes as the target of inference a general parameter associated with a class of processes, rather than the average effect in a set of cases. Here we implement such an analysis, similar to that pre-specified in our MPAP as a secondary analysis. (In the MPAP, we specified an analysis that assesses the distribution of effects based on the count of votes for the incumbent and the total number of voters. The analysis as specified, however, is at odds with the design, since it does not take account of the fact that the treatment was randomized within blocks. Accounting for this would require a more complex multilevel structure with block and country effects; instead we elected to use a closely related model that is similar in spirit but that uses the study-level estimated effects as inputs).

The key feature of the approach is that we assume that the treatment effect in a particular case, i, is drawn from a population of treatment effects with mean μ and standard deviation τ. Note that there is no assumption of homogeneity across cases. If in fact there is large fundamental heterogeneity, then we should infer a large τ. Note also that "fundamental" uncertainty here does not mean that common logics do not obtain across places; it is possible that heterogeneity arises because of other unmodeled features, such as characteristics of subjects or of polities. If modeled, the mean μ could be a function of these features, and we would expect lower values of τ. Given the lack of observed heterogeneity in effects, we do not pursue that approach.

The simplest analysis, which we present here, uses only the information provided above on the estimated effects and estimates of uncertainty (clustered standard errors) for each case, which we will call $\widehat{\mu}_j$ and σ_j. We place flat priors on μ and on τ (subject to a non-negativity constraint), and the likelihood function uses the probability of observing the estimate for a given country $\widehat{\mu}_j$ given σ_j and parameter μ_j, where the probability of μ_j is itself a function of μ and τ

$$\mu_j \sim N(\mu, \tau)$$
$$\widehat{\mu}_j \sim N(\mu_j, \sigma_j)$$

Note that this analysis treats the individual case estimates as if they were drawn from a common distribution. This is clearly a very strong assumption and requires at a minimum a conceptualization of the kinds of cases that form the population as well as an assumption that the selection of a case is not related to the size of its treatment effect. In addition the particular model assumes normality; this is also a substantive assumption, though not as fundamental as the assumption regarding case selection.

Bayesian analysis allows for estimation of the parameters of this model: μ, τ and $\mu_j, j = 1, 2, \ldots 6$. The results are shown in Figure 7 for candidate support for the good news and bad news cases, and Figure 8 for turnout.

We see from these results that the estimated μ is very similar to the estimated average effect in our main frequentist analyses, in all cases very close to zero. We also estimate quite a low level of fundamental heterogeneity, which in general spans zero. Finally, as is typical in such models, we see that our individual estimates for cases are in general closer to our estimate of μ than the estimates generated by each case separately. Note that exceptional cases—for instance, the larger point estimates of good news for the Uganda 1 and Burkina Faso studies—get substantially revised in this meta-analysis, reflecting the singularity of the results but also the fact that they are themselves measured with considerable uncertainty. Results of the meta-analysis for the bad news/turnout case suggest similarly weak effects as the primary frequentist analysis, with the credible interval for the posterior crossing zero.

To further probe the robustness of this result, we also conducted an analysis in which we sequentially leave out one study at a time and estimate μ and τ under this assumption. The analysis confirms that overall results differ little from those in Figure 7 and 8.

Overall, the Bayesian results support the conclusion of our frequentist analysis: effects of the common arm intervention are small, and quite uniformly small, across cases.

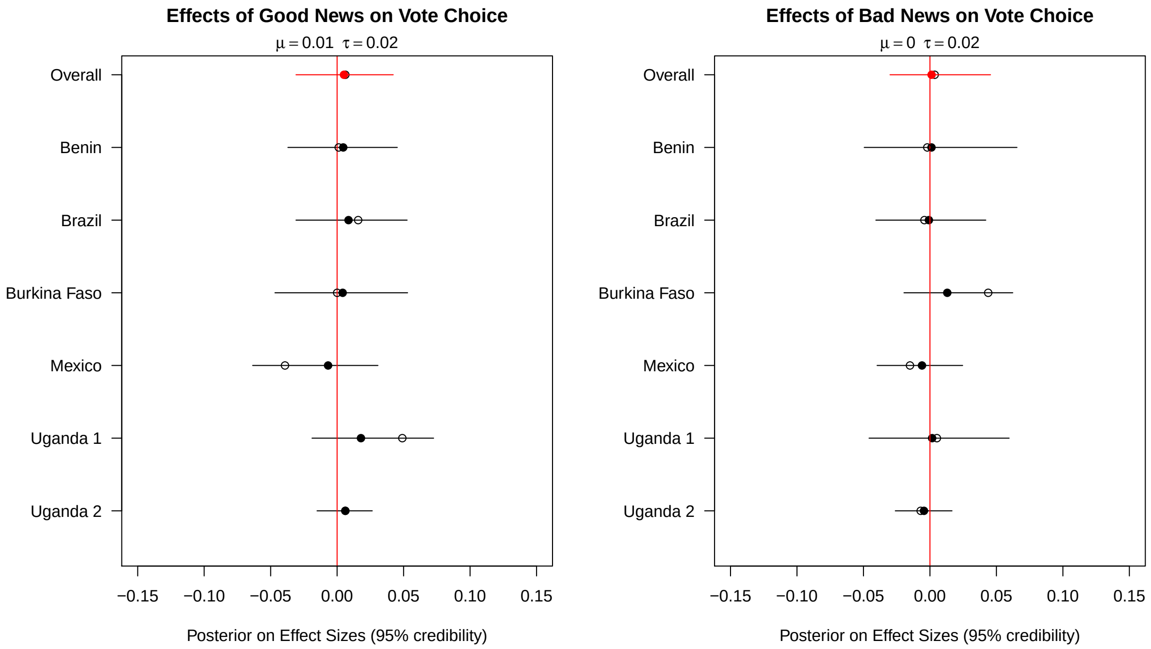

Fig. S7. Bayesian meta-analysis: Vote choice. The solid dots and lines show the estimates from the Bayesian model; the top row shows the overall meta-estimate of μ and τ. The white dots show the original frequentist estimates: in many cases shrinkage can be observed, especially in cases that have effects that are more imprecisely estimated.

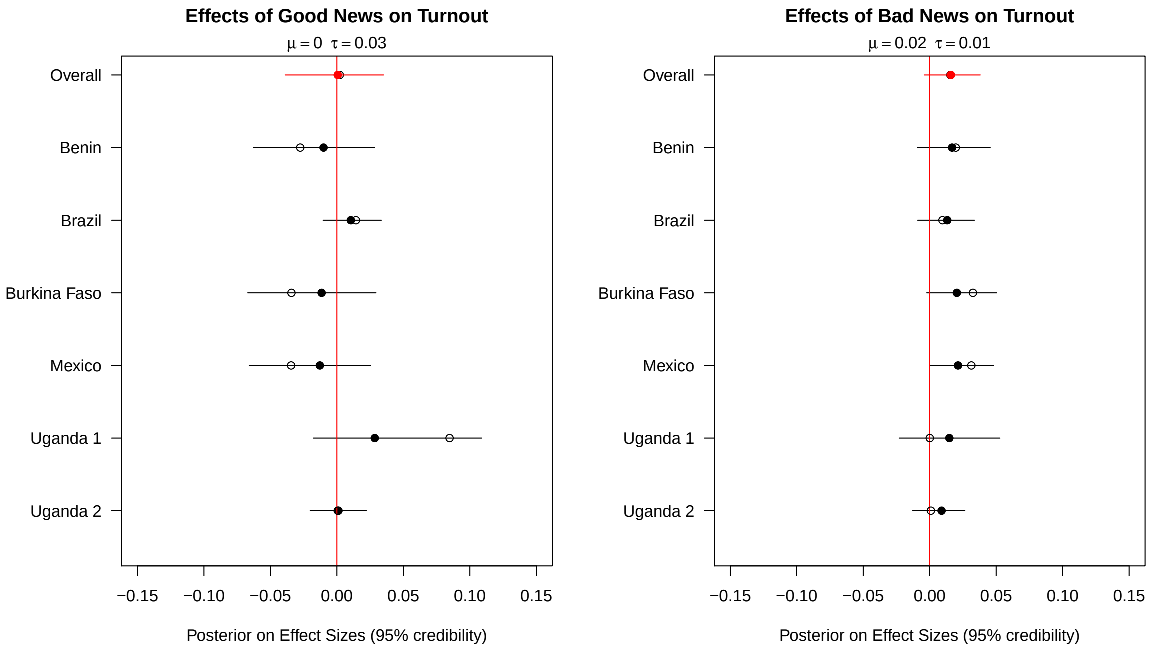

Fig. S8. Bayesian meta-analysis: Turnout. The solid dots and lines show the estimates from the Bayesian model; the top row shows the overall meta-estimate of μ and τ. The white dots show the original frequentist estimates: in many cases shrinkage can be observed, especially in cases that have effects that are more imprecisely estimated.

Section S4. Possible explanations for the null findings

4.1 Voter Updating

4.1.1 Manipulation Check

Table S8. Manipulation check: Effect of treatment on correct recollection, pooling good and bad news (unregistered analysis).

	Correct Recollection					
	Overall	Benin	Brazil	Mexico	Uganda 1	Uganda 2
	(1)	(2)	(3)	(4)	(5)	(6)
Treatment	0.072***	0.050	0.038	0.149***	0.119***	−0.0001
	(0.015)	(0.059)	(0.021)	(0.015)	(0.035)	(0.008)
Covariates	No	No	No	No	No	No
Observations	16,173	897	1,677	2,089	750	10,760
R^2	0.320	0.276	0.378	0.137	0.035	0.205

Notes: The table reports results on manipulation checks across studies, using recollection or accuracy tests at endline that were specific to the content of each study's interventions (MPAP measure M30). The dependent variable, correct recollection, is dichotomized in each study using the following measures: Benin: whether correctly recalled the relative performance of incumbent in plenary and committee work; Brazil: whether correctly recalled whether municipal account was accepted or rejected; Mexico: identification of content of the flyer; Uganda 1: index consisting of knowledge of MP responsibilities, MP priorities for constituency, and identities of contesting candidates. Individuals with an index equal to or greater than 1.5 on a 0-3 scale were coded as correct recalls; Uganda 2: whether correctly recalled relative financial accountability relative to other districts. We include randomization block fixed effects. Standard errors are clustered at the level of treatment assignment. *p<0.05; **p<0.01; ***p<0.001.

Table S9. Manipulation check: Absolute difference between posterior and prior beliefs for pooled good and bad news (unregistered analysis).

	Absolute difference between posterior and prior beliefs			
	Overall	Benin	Brazil	Uganda 2
	(1)	(2)	(3)	(4)
Treatment	0.006	0.063	−0.003	−0.023
	(0.025)	(0.089)	(0.022)	(0.023)
Covariates	No	No	No	No
Observations	12,704	389	1,677	10,638
R^2	0.241	0.176	0.358	0.111

Notes: The table reports differences between beliefs about politician performance after (MPAP measure M30) and prior to treatment (MPAP measure M9). Posterior beliefs are measured using recollection tests at endline specific to the content of each study's intervention. Burkina Faso is excluded because their recollection measure was collected among treated subjects only. Mexico is excluded from results because the study does not contain pre-treatment measures of subjects beliefs. Uganda 1 is not included because the M30 measure is an aggregate measure of subjects' political knowledge and cannot be directly compared with the scale used for measuring priors. We include randomization block fixed effects. Standard errors are clustered at the level of treatment assignment. *p<0.05; **p<0.01; ***p<0.001.

4.1.2 Perceptions

Table S10. Effect of information on perception of importance of politician effort and honesty.

	Effort		Dishonesty	
	Good News	Bad News	Good News	Bad News
	(1)	(2)	(3)	(4)
Treatment effect	−0.014	−0.051	−0.053	0.099
	(0.046)	(0.051)	(0.047)	(0.098)
Control mean	2.449	2.7	2.755	2.724
RI p-value	0.8	0.466	0.36	0.756
Joint RI p-value	0.507		0.292	
Covariates	No	No	No	No
Observations	7,039	5,963	7,278	6,755
R^2	0.253	0.294	0.300	0.231

Note: The table reports the effect of the treatment on voters' perception of how hard-working (MPAP measure M5) and dishonest (MPAP measure M6) the incumbent politician is. We pool Benin, Burkina Faso, Uganda 1, and Uganda 2 in columns (1) and (2), and Benin, Burkina Faso, Mexico, and Uganda 2 in columns (3) and (4). MPAP measures M5 (effort) and M6 (dishonesty). Regressions include randomization block fixed effects; standard errors are clustered at the level of treatment assignment. * $p < 0.05$; ** $p < 0.01$; *** $p < 0.001$

Table S11. Effect of information and source credibility on evaluation of politician effort and honesty (unregistered analysis).

	Dependent variable:			
	Effort		Dishonesty	
	Good News	Bad News	Good News	Bad News
	(1)	(2)	(3)	(4)
Treatment	−0.034	−0.088	−0.037	−0.037
	(0.079)	(0.090)	(0.085)	(0.085)
Credible Source	−0.051	−0.010	−0.022	−0.022
	(0.079)	(0.081)	(0.064)	(0.064)
Treatment * Credible Source	0.033	0.070	0.010	0.010
	(0.095)	(0.105)	(0.093)	(0.093)
Control mean	2.451	2.703	2.75	2.75
RI p-values	0.725	0.516	0.72	0.72
Joint RI p-value	0.476		0.72	
Covariates	No	No	No	No
Observations	6,436	5,406	6,483	6,483
R^2	0.261	0.293	0.329	0.329

Note: The table reports the effects of information and the credibility of the information source on voter's perception of how hard-working (MPAP measure M5) and dishonest (MPAP measure M6) the incumbent politician is. We pool Benin, Burkina Faso, Uganda 1, and Uganda 2 in columns (1) and (2), and Benin, Burkina Faso, Mexico, and Uganda 2 in columns (3) and (4). Regressions include randomization block fixed effects; standard errors are clustered at the level of treatment assignment. * $p < 0.05$; ** $p < 0.01$; *** $p < 0.001$

4.1.3 Association of Perceptions and Electoral Support

Table S12. Relationship between evaluation of politician effort and honesty with vote choice (unregistered analysis).

	Incumbent vote choice			
	Good news		Bad news	
	(1)	(2)	(3)	(4)
Effort	0.052^{***}		0.066^{***}	
	(0.006)		(0.006)	
Dishonesty		-0.054^{***}		-0.026^{***}
		(0.005)		(0.005)
Covariates	No	No	No	No
Observations	11,040	11,452	10,190	10,943
R^2	0.229	0.217	0.282	0.266

Note: The table reports the effects of information and the credibility of the information source on voter's perception of how hard-working (MPAP measure M5) and dishonest (MPAP measure M6) the incumbent politician is. We pool Benin, Burkina Faso, Uganda 1, and Uganda 2 in columns (1) and (3), and Benin, Burkina Faso, Mexico, and Uganda 2 in columns (2) and (4). Results exclude non-contested seats and include vote choice for LCV councilors as well as chairs in the Uganda 2 study. Regressions include randomization block fixed effects; standard errors are clustered at the level of treatment assignment. * $p < 0.05$; ** $p < 0.01$; *** $p < 0.001$

4.2 Politician Response

Table S13. Effect of bad news on politician backlash.

	Politician response / backlash		
	Overall	Benin	Mexico
	(1)	(2)	(3)
Treatment effect	0.069*	0.068	0.070***
	(0.028)	(0.057)	(0.010)
Control mean	0.108	0.068	0.146
RI p-value	0.089	0.438	0
Covariates	No	No	No
Observations	2,052	702	1,350
R^2	0.623	0.504	0.848

Note: The table reports on whether the treatment led to the incumbent party or candidate campaigning on dimensions of the disseminated information (MPAP measure M8). Backlash was measured for studies with clustered assignment. Regressions include randomization block fixed effects; standard errors are clustered at the level of treatment assignment. * $p < 0.05$; ** $p < 0.01$; *** $p < 0.001$

4.3 Learning from Variation

We pre-registered a number of additional hypotheses is the MPAP. We list these in Table 14. Subsequent tables report the results of the additional analysis.

Table S14. Additional hypotheses and results.

MPAP hypothesis	Prediction	Moderator measure	News subgroup	Evidence for interaction
**Substitution effects**: Ethnicity, partisanship, or clientelist relations could provide heuristic substitutes for information				
H6: Non-coethnics	Good news effects more positive for incumbent's non-coethnics	M15	Good	No (Table 15)
H6: Non-coethnics	Bad news effects more negative for incumbent's non-coethnics	M15	Bad	No (Table 15)
H7: Partisanship	Good news effects more positive for voters with weaker partisan identities	M19	Good	No (Table 15)
H7: Partisanship	Bad news effects more negative for voters with weaker partisan identities	M19	Bad	No (Table 15)
H8: Clientelism	Good news effects more positive for voters who have not received clientelistic benefits	M22	Good	No (Table 15)
H8: Clientelism	Bad news effects more negative for voters who have not received clientelistic benefits	M22	Bad	No (Table 15)
**Context-specific heterogeneity**: Information will have greater impact among voters with less exposure to information in the pre-treatment period, and in competitive, free and fair elections				
H9: Informational environment	Good news effects are more positive in low information environments	M11	Good	No (Table 16)
H9: Informational environment	Bad news effects are more negative in low information environments	M11	Bad	No (Table 16)
H10: Competitive elections	Good news effects are more positive where electoral competition is greater	M25	Good	No (Table 17)
H10: Competitive elections	Bad news effects are more negative where electoral competition is greater	M25	Bad	No (Table 17)
H11: Free and fair elections	Good news effects are more positive where elections are believed to be free and fair	M26/M27	Good	No (Table 16)
H11: Free and fair elections	Bad news effects are more negative where elections are believed to be free and fair	M26/M27	Bad	No (Table 16)
**Intervention-specific heterogeneity**				
H12: Information content	Information effects—both positive and negative—are stronger when the gap between voters' prior beliefs about candidates and the information provided is larger	N_{ij}	All	No (Table 18)
H13: Information welfare salient	Good news effects are more positive the more the information relates directly to individual welfare	M23	Good	No (Table 18)
H13: Information welfare salient	Bad news effects are more negative the more the information relates directly to individual welfare	M23	Bad	No (Table 18)
H14: Credible source	Good news effects are more positive the more reliable and credible is the information source	M24	Good	No (Table 18)
H14: Credible source	Bad news effects are more negative the more reliable and credible is the information source	M24	Bad	No (Table 18)
Covariate-treatment interactions in MPAP equations (3) and (4)		Demographics		No (Tables 19 and 20)

4.3.1 Substitution Effects

Table S15. Effect of moderators on incumbent vote choice.

	Incumbent vote choice					
	Good news	Bad news	Good news	Bad news	Good news	Bad news
	(1)	(2)	(3)	(4)	(5)	(6)
Treatment	0.018	0.0004	−0.0001	0.013	0.001	0.004
	(0.015)	(0.022)	(0.025)	(0.021)	(0.014)	(0.016)
Coethnicity	−0.022	0.0003				
	(0.029)	(0.041)				
Treatment * Coethnicity	0.058	−0.042				
	(0.033)	(0.049)				
Copartisanship			0.216***	0.289***		
			(0.032)	(0.028)		
Treatment * Copartisanship			0.001	0.004		
			(0.038)	(0.036)		
Clientelism					−0.041***	−0.044***
					(0.009)	(0.011)
Treatment * Clientelism					0.013	0.006
					(0.012)	(0.015)
Control mean	0.365	0.442	0.36	0.397	0.359	0.383
RI p-values	0.297	0.989	0.998	0.564	0.938	0.814
Joint RI p-value	0.628		0.826		0.86	
Covariates	No	No	No	No	No	No
Observations	11,502	10,320	11,688	10,999	13,246	12,288
R^2	0.268	0.230	0.276	0.289	0.279	0.259

Note: The table reports results of the treatment on three pre-specified moderators—coethnicity (MPAP measure M15), copartisanship (MPAP measure M19) and indulging in clienetelistic practices (MPAP measure M22)—on incumbent vote choice. The following cases are included in each regression: Co-ethnicity—Benin, Brazil, Uganda 1, Uganda 2; Co-partisanship—Benin, Brazil, Mexico, Uganda 1, Uganda 2; Clientelism—Benin, Burkina Faso, Brazil, Mexico, Uganda 1, Uganda 2. Pooled results exclude non-contested seats and include vote choice for LCV councilors as well as chairs in the Uganda 2 study. Regressions include randomization block fixed effects; standard errors are clustered at the level of treatment assignment. * $p < 0.05$; ** $p < 0.01$; *** $p < 0.001$

4.3.2 Context-Specific Heterogeneity

Table S16. Effect of information and context heterogeneity on incumbent vote choice.

	Incumbent vote choice					
	Good news	Bad news	Good news	Bad news	Good news	Bad news
	(1)	(2)	(3)	(4)	(5)	(6)
Treatment	−0.062	−0.011	0.015	−0.005	−0.030	0.019
	(0.055)	(0.054)	(0.024)	(0.030)	(0.034)	(0.034)
Certainty	−0.015	0.021				
	(0.017)	(0.018)				
Treatment * Certainty	0.032	−0.003				
	(0.024)	(0.024)				
Secret ballot			−0.001	0.010		
			(0.008)	(0.010)		
Treatment * Secret ballot			−0.005	0.005		
			(0.010)	(0.011)		
Free, fair election					−0.001	0.005
					(0.008)	(0.009)
Treatment * Free, fair election					0.012	−0.004
					(0.010)	(0.011)
Control means	0.362	0.412	0.383	0.357	0.352	0.386
RI p-values	0.281	0.858	0.572	0.874	0.423	0.588
Joint RI p-value	0.412		0.676		0.326	
Covariates	No	No	No	No	No	No
Observations	10,993	9,622	13,419	12,589	13,111	12,422
R^2	0.328	0.267	0.258	0.235	0.262	0.239

Note: The table reports results of whether the treatment had different effects depending on voters' certainty about their priors (MPAP measure M11), and their perceptions about the secrecy of their ballot (MPAP measure M26) and how free and fair the election was (MPAP measure M27). Pooled results exclude non-contested seats and include vote choice for LCV councilors as well as chairs in the Uganda 2 study. Regressions include randomization block fixed effects; standard errors are clustered at the level of treatment assignment. * $p < 0.05$; ** $p < 0.01$; *** $p < 0.001$

Table S17. Effect of information and electoral competition on vote choice.

	Incumbent vote choice			
	Low competition		High competition	
	Good news	Bad news	Good news	Bad news
	(1)	(2)	(3)	(4)
Treatment	0.009	−0.043	0.004	0.015
	(0.022)	(0.031)	(0.030)	(0.037)
Control mean	0.342	0.414	0.392	0.294
RI p-values	0.716	0.254	0.904	0.746
Covariates	No	No	No	No
Observations	1,450	1,433	1,113	1,307
R^2	0.221	0.231	0.240	0.128

Note: The table reports results of whether the treatment had different effects in constituencies with low or high levels of electoral competition (MPAP measure M25). We pool Benin, Brazil, Mexico, and Uganda 1. Regressions include randomization block fixed effects; standard errors are clustered at the level of treatment assignment. $^{*}\ p < 0.05$; $^{**}\ p < 0.01$; $^{***}\ p < 0.001$

4.3.3 Intervention-Specific Heterogeneity

Table S18. Effect of information and intervention-specific heterogeneity on vote choice.

	Incumbent vote choice					
	Good news	Bad news	Good news	Bad news	Good news	Bad news
	(1)	(2)	(3)	(4)	(5)	(6)
Treatment	0.001	−0.010	0.025	−0.022	−0.017	−0.013
	(0.016)	(0.016)	(0.024)	(0.036)	(0.021)	(0.023)
N_{ij}	−0.027	−0.053***				
	(0.016)	(0.014)				
Treatment * N_{ij}	−0.006	−0.006				
	(0.020)	(0.019)				
Information salient			−0.016	−0.041		
			(0.029)	(0.035)		
Treatment * Information salient			−0.015	0.053		
			(0.034)	(0.042)		
Credible source					−0.007	0.005
					(0.028)	(0.027)
Treatment * Credible source					0.036	0.020
					(0.030)	(0.031)
Control mean	0.356	0.398	0.355	0.435	0.363	0.385
RI p-values	0.956	0.592	0.314	0.61	0.452	0.628
Joint RI p-value	0.779		0.232		0.347	
Covariates	No	No	No	No	No	No
Observations	13,274	12,563	12,343	10,587	12,354	11,407
R^2	0.275	0.249	0.265	0.221	0.260	0.240

Note: The table reports results of the effect of information and (a) the gap between priors and information (MPAP measure N_{ij}), (b) salience of information (MPAP measure M23) and (c) credibility of information source on voters' decision to vote for the incumbent. Columns 1, 2, 5 and 6 pool observations from all studies while Columns 3 and 4 pool Benin, Brazil, Uganda 1 and Uganda 2. Results exclude non-contested seats and include vote choice for LCV councilors as well as chairs in the Uganda 2 study. Regressions include randomization block fixed effects; standard errors are clustered at the level of treatment assignment. * $p < 0.05$; ** $p < 0.01$; *** $p < 0.001$

4.3.4 Heterogeneity by Demographics

Table S19. Interaction analysis: Effect of good news on incumbent vote choice.

	Incumbent vote choice, good news						
	ALL	BEN	BRZ	BF	MEX	UG 1	UG 2
	(1)	(2)	(3)	(4)	(5)	(6)	(7)
Treatment	0.0002	−0.033	0.007	0.004	−0.036	0.048	0.009
	(0.015)	(0.065)	(0.030)	(0.049)	(0.031)	(0.033)	(0.012)
N_{ij}	−0.015	0.008		−0.016		−0.052**	−0.010
	(0.016)	(0.062)	(0.000)	(0.039)	(0.000)	(0.018)	(0.009)
Treatment * N_{ij}	0.001	0.131	−0.026	−0.028	0.034	−0.025	−0.003
	(0.008)	(0.077)	(0.023)	(0.056)	(0.018)	(0.013)	(0.006)
Age	−0.001	−0.008	0.001	0.002	−0.003	0.003*	0.002**
	(0.001)	(0.006)	(0.002)	(0.003)	(0.002)	(0.002)	(0.001)
Treatment * Age	−0.005	−0.140	−0.060*	−0.083*	0.054**	−0.009	0.004
	(0.008)	(0.072)	(0.024)	(0.037)	(0.020)	(0.015)	(0.006)
Education	−0.002	−0.009	0.010	0.002	−0.003	−0.011	−0.002
	(0.003)	(0.010)	(0.007)	(0.020)	(0.008)	(0.007)	(0.003)
Treatment * Education	−0.012	−0.037		−0.018		0.035	−0.013
	(0.020)	(0.068)	(0.000)	(0.050)	(0.000)	(0.027)	(0.012)
Wealth	0.022	0.038	0.061	−0.007	0.033	0.041	0.016
	(0.013)	(0.049)	(0.039)	(0.039)	(0.034)	(0.027)	(0.009)
Treatment * Wealth	0.001	0.014*	−0.004	0.001	0.004	−0.005*	0.0004
	(0.001)	(0.007)	(0.003)	(0.005)	(0.003)	(0.002)	(0.001)
Voted previously	0.053	−0.070	0.073	0.096	0.185***	−0.157**	0.057*
	(0.027)	(0.068)	(0.079)	(0.085)	(0.048)	(0.057)	(0.025)
Treatment * Voted previously	0.008	0.025	−0.010	−0.033	0.009	0.019*	−0.003
	(0.004)	(0.016)	(0.008)	(0.026)	(0.010)	(0.009)	(0.003)
Supported incumbent	0.191***	0.004	0.293***	0.242	0.308***	0.178**	0.111***
	(0.028)	(0.107)	(0.058)	(0.147)	(0.049)	(0.055)	(0.024)
Treatment * Supported incumbent	−0.041*	−0.134	0.030	0.036	−0.079	−0.129**	0.003
	(0.018)	(0.086)	(0.052)	(0.052)	(0.046)	(0.041)	(0.012)
Clientelism	−0.040***	−0.096	−0.073***	0.007	−0.054*	−0.019	−0.006
	(0.010)	(0.077)	(0.021)	(0.086)	(0.026)	(0.018)	(0.006)
Treatment * Clientelism	−0.030	0.149	0.085	−0.053	−0.156*	0.026	0.041
	(0.039)	(0.133)	(0.110)	(0.125)	(0.071)	(0.086)	(0.034)
Credible source	−0.022	−0.123	0.025	−0.089	−0.008	−0.052	−0.0001
	(0.033)	(0.172)	(0.112)	(0.081)	(0.065)	(0.049)	(0.032)
Treatment * Credible source	−0.030	−0.129	0.092	−0.006	0.112	−0.109	−0.002
	(0.041)	(0.110)	(0.073)	(0.197)	(0.093)	(0.075)	(0.033)
Secret ballot	0.016	0.143	−0.016	0.100	0.042	0.009	0.007
	(0.013)	(0.112)	(0.027)	(0.123)	(0.035)	(0.023)	(0.009)
Treatment * Secret ballot	0.058	0.283	−0.042	0.052	0.120	0.074	0.011
	(0.044)	(0.247)	(0.137)	(0.124)	(0.086)	(0.068)	(0.043)
Free, fair election	−0.002	−0.121	0.012	0.003	−0.036	0.040*	−0.004
	(0.011)	(0.083)	(0.031)	(0.076)	(0.028)	(0.019)	(0.008)
Treatment * free, fair election	0.019	0.022	0.020	0.117*	−0.016	0.030	0.008
	(0.012)	(0.082)	(0.026)	(0.050)	(0.032)	(0.022)	(0.008)
Covariates	Yes	Yes	Yes	Yes	Yes	Yes	Yes
Observations	13,190	214	859	389	725	456	10,547
R^2	0.298	0.360	0.484	0.392	0.224	0.177	0.240

Note: The table presents results from fitting MPAP equation (3). Standard errors are clustered at the level of treatment assignment. Results in columns (1) and (7) include vote choice for LCV councilors as well as chairs in the Uganda 2 study (see Buntaine et al., Chapter 8). This means each respondent in the Uganda 2 study enters twice, and we cluster the standard errors at the individual level. * $p < 0.05$; ** $p < 0.01$; *** $p < 0.001$

Table S20. Interaction analysis: Effect of bad news on incumbent vote choice.

	Incumbent vote choice, bad news						
	ALL	BEN	BRZ	BF	MEX	UG 1	UG 2
	(1)	(2)	(3)	(4)	(5)	(6)	(7)
Treatment	−0.003	−0.079	−0.022	0.037	−0.013	0.010	−0.006
	(0.015)	(0.086)	(0.030)	(0.028)	(0.018)	(0.053)	(0.012)
N_{ij}	−0.049***	−0.091	−0.100***	−0.005		−0.036	−0.002
	(0.015)	(0.048)	(0.028)	(0.026)	(0.000)	(0.036)	(0.009)
Treatment * N_{ij}	−0.001	−0.117	−0.004	0.018	0.047***	−0.015	−0.002
	(0.011)	(0.094)	(0.021)	(0.033)	(0.013)	(0.028)	(0.006)
Age	0.0004	−0.004	0.00003	0.002	0.0003	0.002	0.001
	(0.001)	(0.004)	(0.002)	(0.002)	(0.001)	(0.003)	(0.001)
Treatment * Age	0.0002	−0.015	0.001	−0.030	0.012	0.019	−0.003
	(0.011)	(0.072)	(0.026)	(0.020)	(0.013)	(0.032)	(0.007)
Education	−0.003	−0.006	−0.001	−0.006	−0.010*	0.0004	−0.003
	(0.003)	(0.010)	(0.005)	(0.008)	(0.004)	(0.012)	(0.003)
Treatment * Education	−0.001	0.090	−0.063	−0.004		−0.002	−0.003
	(0.020)	(0.070)	(0.034)	(0.031)	(0.000)	(0.053)	(0.012)
Wealth	0.037*	0.021	0.012	0.001	0.037	0.089	0.013
	(0.015)	(0.093)	(0.041)	(0.023)	(0.020)	(0.045)	(0.009)
Treatment * Wealth	−0.00005	0.003	−0.001	0.001	0.0002	−0.001	−0.001
	(0.001)	(0.008)	(0.002)	(0.002)	(0.001)	(0.004)	(0.001)
Voted previously	0.036	0.063	−0.053	0.083	0.123**	−0.138	0.075*
	(0.037)	(0.282)	(0.089)	(0.047)	(0.038)	(0.103)	(0.029)
Treatment * Voted previously	0.001	−0.013	0.006	0.006	−0.005	−0.003	0.002
	(0.005)	(0.018)	(0.007)	(0.012)	(0.006)	(0.017)	(0.004)
Supported incumbent	0.190***	−0.021	0.282***	0.249***	0.465***	0.204*	0.065*
	(0.046)	(0.165)	(0.049)	(0.067)	(0.035)	(0.090)	(0.030)
Treatment * Supported incumbent	−0.025	0.025	0.027	−0.018	0.015	−0.105	−0.024
	(0.020)	(0.104)	(0.054)	(0.031)	(0.033)	(0.061)	(0.012)
Clientelism	−0.032**	−0.011	−0.086***	0.019	−0.012	−0.020	0.005
	(0.010)	(0.139)	(0.019)	(0.055)	(0.016)	(0.026)	(0.007)
Treatment * Clientelism	−0.023	−0.276	0.186	−0.042	0.030	−0.094	0.027
	(0.046)	(0.313)	(0.105)	(0.066)	(0.052)	(0.145)	(0.041)
Credible source	−0.012	−0.055	0.015	−0.042	0.027	−0.025	0.002
	(0.034)	(0.205)	(0.075)	(0.051)	(0.040)	(0.083)	(0.036)
Treatment * Credible source	0.016	−0.104	−0.052	0.133	−0.090	0.073	0.011
	(0.055)	(0.236)	(0.068)	(0.086)	(0.058)	(0.116)	(0.042)
Secret ballot	−0.008	−0.032	0.026	0.027	−0.056*	−0.013	−0.007
	(0.014)	(0.144)	(0.025)	(0.076)	(0.023)	(0.037)	(0.009)
Treatment * Secret ballot	0.049	0.283	0.029	0.095	0.012	−0.015	0.056
	(0.047)	(0.486)	(0.114)	(0.081)	(0.056)	(0.115)	(0.051)
Free, fair election	0.016	−0.001	0.043	0.013	−0.033	0.033	−0.003
	(0.014)	(0.211)	(0.027)	(0.043)	(0.018)	(0.037)	(0.009)
Treatment * free, fair election	−0.004	0.154	−0.018	0.003	−0.015	−0.039	0.010
	(0.014)	(0.150)	(0.029)	(0.029)	(0.024)	(0.045)	(0.010)
Covariates	Yes	Yes	Yes	Yes	Yes	Yes	Yes
Observations	12,531	181	818	911	1,215	294	9,112
R^2	0.281	0.306	0.420	0.311	0.296	0.208	0.278

Note: The table presents results from fitting MPAP equation (4). Standard errors are clustered at the level of treatment assignment. Results in columns (1) and (7) include vote choice for LCV councilors as well as chairs in the Uganda 2 study (see Buntaine et al., Chapter 8). This means each respondent in the Uganda 2 study enters twice, and we cluster the standard errors at the individual level. * $p < 0.05$; ** $p < 0.01$; *** $p < 0.001$

Section S5. Effects of publicly disseminated information

Table S21. Private versus public information: Effect of good news on incumbent vote choice.

	Incumbent vote choice, good news			
	Overall	Benin	Mexico	Uganda 1
	(1)	(2)	(3)	(4)
Private information	−0.008	0.012	−0.029	0.008
	(0.023)	(0.044)	(0.043)	(0.027)
Public information	0.055*	0.146**	−0.002	0.019
	(0.022)	(0.047)	(0.041)	(0.023)
Control mean	0.356	0.439	0.498	0.186
F-test p-value	0.018	0.006	0.598	0.708
Covariates	No	No	No	No
Observations	2,962	776	784	1,402
R^2	0.192	0.189	0.088	0.068

Note: The table reports results of the effect of good news about the incumbent on vote choice, depending on whether voters received this information in private or public settings. We pool Benin, Mexico, and Uganda 1. Regressions include randomization block fixed effects and standard errors are clustered at the level of treatment assignment. * $p < 0.05$; ** $p < 0.01$; *** $p < 0.001$

Table S22. Private versus public information: Effect of bad news on incumbent vote choice.

	Incumbent vote choice, bad news			
	Overall	Benin	Mexico	Uganda 1
	(1)	(2)	(3)	(4)
Private information	−0.027	−0.012	−0.036	−0.035
	(0.030)	(0.074)	(0.030)	(0.042)
Public information	0.009	0.006	0.015	0.009
	(0.026)	(0.069)	(0.032)	(0.032)
Control mean	0.441	0.535	0.383	0.426
F-test p-value	0.018	0.006	0.598	0.708
Covariates	No	No	No	No
Observations	2,909	601	1,309	999
R^2	0.178	0.241	0.102	0.153

Note: The table reports results of the effect of bad news about the incumbent on vote choice, depending on whether voters received this information in private or public settings. We pool Benin, Mexico, and Uganda 1. Regressions include randomization block fixed effects and standard errors are clustered at the level of treatment assignment. * $p < 0.05$; ** $p < 0.01$; *** $p < 0.001$
